# Phase-shifting optothermal microscopy enables live-cell mid-infrared hyperspectral imaging of large cell populations at high confluency

**DOI:** 10.1126/sciadv.adj7944

**Published:** 2024-02-21

**Authors:** Tao Yuan, Lucas Riobo, Francesca Gasparin, Vasilis Ntziachristos, Miguel A. Pleitez

**Affiliations:** ^1^ Chair of Biological Imaging at the Central Institute for Translational Cancer Research (TranslaTUM), School of Medicine and Health, Technical University of Munich, Munich, Germany.; ^2^ Institute of Biological and Medical Imaging, Helmholtz Zentrum München, Neuherberg, Germany.; ^3^ Munich Institute of Biomedical Engineering (MIBE), Technical University of Munich, Garching b. München, Germany.

## Abstract

Rapid live-cell hyperspectral imaging at large fields of view (FOVs) and high cell confluency remains challenging for conventional vibrational spectroscopy–based microscopy technologies. At the same time, imaging at high cell confluency and large FOVs is important for proper cell function and statistical significance of measurements, respectively. Here, we introduce phase-shifting mid-infrared optothermal microscopy (PSOM), which interprets molecular-vibrational information as the optical path difference induced by mid-infrared absorption and can take snapshot vibrational images over broad excitation areas at high live-cell confluency. By means of phase-shifting, PSOM suppresses noise to a quarter of current optothermal microscopy modalities to allow capturing live-cell vibrational images at FOVs up to 50 times larger than state of the art. PSOM also reduces illumination power flux density (PFD) down to four orders of magnitude lower than other conventional vibrational microscopy methods, such as coherent anti-Stokes Raman scattering (CARS), thus considerably decreasing the risk of cell photodamage.

## Teaser

PSOM achieves wide-field label-free chemical imaging with enhanced MIR-phase sensitivity in high-confluence live-cell populations.

## INTRODUCTION

Vibrational spectroscopy–based microscopy methods, such as coherent anti-Stokes Raman scattering (CARS) (*1*, *2*), stimulated Raman scattering (SRS) (*3*), and mid-infrared (mid-IR) microscopy (*4*), are label-free imaging modalities that take advantage of the bond-specific vibrational transitions of biomolecules to achieve intrinsic chemical contrast in living cells and tissues. In particular, in vibrational microscopy, hyperspectral images (where each pixel comprises a broad vibrational spectrum) can be acquired to allow the differentiation of biomolecule subgroups—i.e., to discriminate among different types of lipids, proteins, and carbohydrates by computational analysis of hyperspectral images (*5*, *6*). In this way, label-free chemical microscopy could be applied for live-cell metabolic imaging—avoiding the use of external fluorescent labels needed in conventional florescence microscopy, which can undergo photobleaching (*7*) and photoconversion (*8*) and possibly alter the metabolic activity of cells (*9*). However, most vibrational imaging methods apply tightly focused excitation and point-by-point raster scanning for image formation, which results in slow spectral-imaging speed particularly at large fields of view (FOVs). For instance, in mid-IR hyperspectral imaging, using quantum cascade lasers for excitation and mechanical scanning for image formation results in spectral-imaging times on the order of minutes to hours—precluding live-cell hyperspectral imaging and increasing the risk of cell photodamage due to long exposures (*10*). Raman imaging, in combination with beam scanning using galvo mirrors, can reach video rate imaging speeds (*11*); however, this approach is restricted to small FOVs (~100 × 100 μm) requiring imaging stitching to achieve larger FOVs (*6*, *12*). In addition, although wide-field Fourier transform infrared (FTIR) microscopy has been developed to increase hyperspectral imaging speed (*13*), this approach has been mainly applied to dry thin tissue sections, due to reduced applicability for live-cell microscopy because of the strong mid-IR absorption of water required in cell cultures.

To increase imaging speed—profiting from mid-IR absorption with cross sections eight orders of magnitude larger than Raman scattering—wide-field mid-IR microscopy methods based on the optothermal effect and optical phase-contrast detection for rapid hyperspectral imaging have been recently proposed (*14**–**16*). For instance, Zhang *et al.* (*14*) developed bond-selective transient phase (BSTP) imaging, in which mid-IR excitation results in a transient refractive index change in the excited sample that is detected by a phase-contrast microscope. BSTP enabled hyperspectral imaging in living cells at above-video-rate speed, i.e., above 24 frames per second. Similarly, Tamamitsu *et al.* (*15*) developed molecular vibration-sensitive quantitative phase-contrast imaging (MV-QPI), which detects mid-IR absorption images by quantitative phase-contrast detection. Just like BSTP, MV-QPI has also shown above-video-rate label-free mid-IR hyperspectral imaging in living HeLa cells with subcellular spatial resolution. However, despite offering high-imaging speed and demonstrations of their feasibility for live-cell hyperspectral imaging, BSTP and MV-QPI are restricted to small FOVs of 10 to 50 μm, whereas FOVs above 100 μm are necessary for imaging cell populations. To address the limitation in FOV size, Yuan *et al.* (*16*) recently introduced wide-field optothermal mid-IR microscopy (WOMiM) based on snapshot pump-probe detection of the optothermal signal. With WOMiM, the authors demonstrated chemical contrast imaging of an unprecedentedly wide FOV of up to 180 μm, achieving hyperspectral imaging of lipids [triglycerides (TAGs)] in the 2950 to 2830 cm^−1^ range. However, due to low sensitivity, the working principle of WOMiM was demonstrated only on synthetic TAG phantoms.

In wide-field optothermal imaging, phase difference (termed MIR phase) micrographs induced by mid-IR absorption are obtained by subtracting a strong phase background (termed intrinsic phase) caused by a sample’s intrinsic optical properties (e.g., refractive index). The quantitative intrinsic-phase image is usually removed using postprocessing methods such as the Hilbert transform (*17*), derivative method (*18*), and nonlinear phase unwrapping method (*19*). However, because the MIR phase is two to three orders of magnitude smaller than the intrinsic phase (*14*, *20*), these postprocessing methods usually alter the weak MIR-phase signal, resulting in poor detection sensitivity. Although adaptive dynamic range shift (ADRIFT) quantitative phase imaging has been recently developed to bridge the large phase gap between the intrinsic phase and the MIR phase (*20*), this method has not yet been demonstrated at large FOVs for imaging live-cell populations.

As a step forward in wide-field mid-IR optothermal microscopy, we introduce here phase-shifting optothermal microscopy (PSOM), which implements wide-field excitation for a large FOV and incorporates a polarization-based phase-shifting module (PSM) to obtain chemical contrast images with high sensitivity despite large intrinsic phases. We hypothesized that achieving intrinsic-phase–independent optothermal imaging will provide high sensitivity to small MIR-phase variations, thus allowing further expansion of the mid-IR beam for a larger FOV, and thereby reducing the mid-IR phototoxicity to cells. Here, we demonstrate how the PSM enables PSOM to reach shot noise sensitivity limits and effectively reduces noise-equivalent phase down to 0.26 mrad, which is a quarter of the state of the art (*20*). Low noise allows PSOM to sense a small MIR-phase variation under a wide-field and low power flux density (PFD) excitation. In our operational examples, PSOM achieved an unprecedentedly large FOV (50 times larger in area than state-of-the-art FOV) (*21*) for live-cell mid-IR optothermal imaging, using excitation PFD four orders of magnitude below the values typically used in conventional vibrational imaging modalities such as CARS (*22*). As examples of operation, we performed measurements of lipid droplets (LDs) in matured adipocytes at high confluence (100%), which is unprecedented in wide-field photothermal microscopy due to the strong intrinsic phase of highly confluent cells.

## RESULTS

### Basic working principle of PSOM

**Fig. 1.**
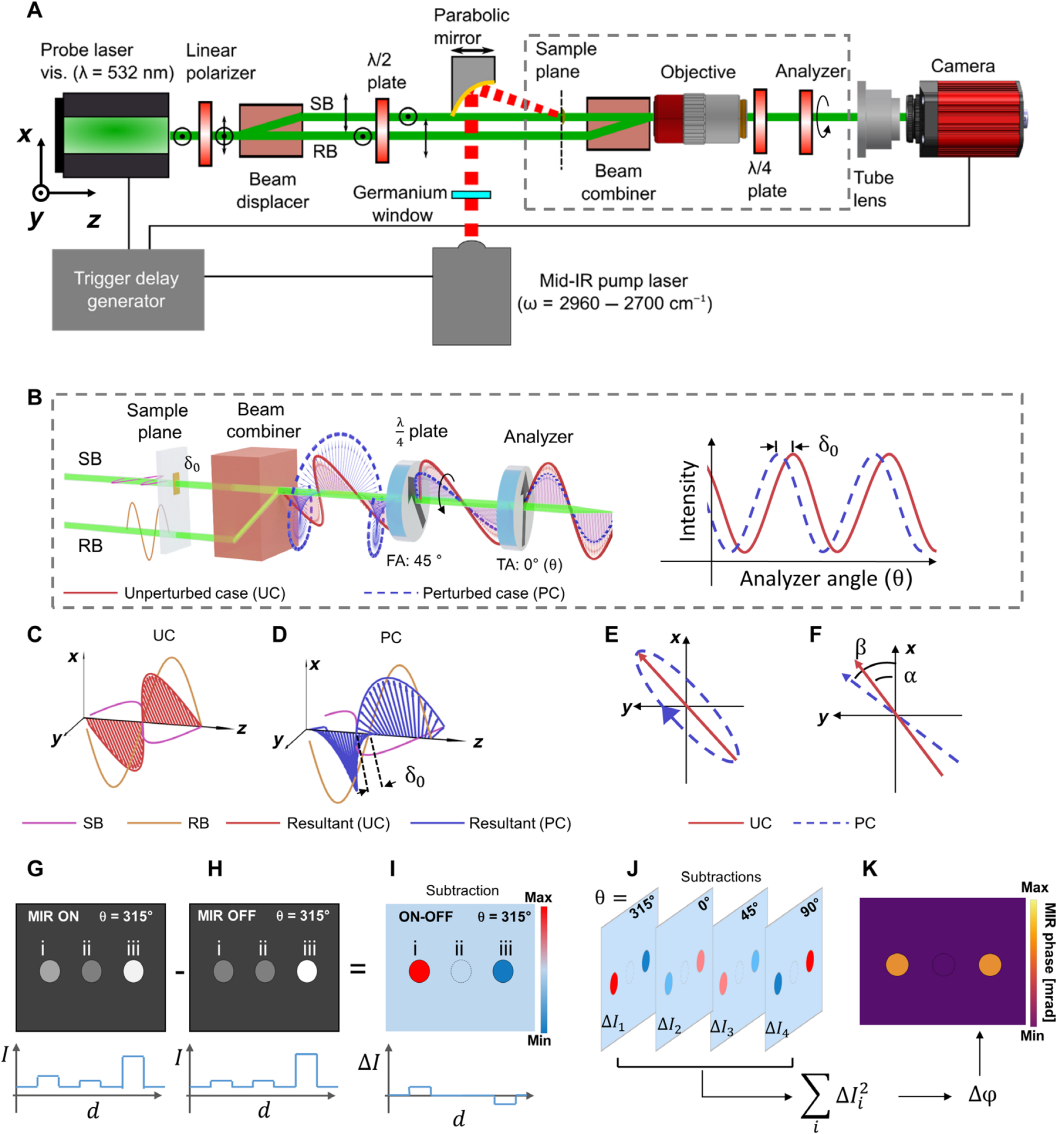
Imaging principle of PSOM. (**A**) Diagram of the overall system. PSOM is a synchronized pump-and-probe imaging method where the PSM probes the phase difference resulting from a wide-field mid-IR excitation (pump) to obtain a chemical contrast image. SB, sample beam; RB, reference beam. (**B**) A phase perturbation δ_0_ in the sample plane results in elliptically polarized light after the beam combiner and rotation of the polarization plane of the beam after the quarter-wave plate. The rotation of the polarization plane is analyzed by changing the transmission axis (TA) of the analyzer. FA, fast axis. The objective shown in (A) is removed (B) to emphasize beam polarization. (**C** and **D**) Polarization states of the beams after the beam combiner. (C) Linear polarization is constructed in the unperturbed case (UC), and (D) elliptical polarization is constructed in the perturbed case (PC). (**E**) Polarization states of (C) and (D) illustrated in the *x-y* plane. (**F**) Polarization states of the beam after the λ/4 plate. α, polarization angle in unperturbed case; β, polarization angle in perturbed case. (**G** to **I**) Demonstration of MIR-ON and MIR-OFF subtraction at an analyzer angle of 315°, with a line profile crossing three targets. i, absorption target; ii, nonabsorption target; iii, absorption target at high dynamic range. *I*, intensity; *d*, distance. (**J**) Four subtraction images are obtained at four analyzer angles (θ = 315°, 0°, 45°, and 90°). (**K**) A PSOM image constructed by the square sum ( $\sum_{i}∆I_{i}^{2}$ ) of the subtraction frames illustrates quantitative phase perturbation (∆φ) induced by mid-IR excitation, independently of the intrinsic phase.

Figure 1 schematically depicts the working principle of pump-and-probe PSOM. PSOM is a label-free bond-selective imaging method based on wide-field mid-IR excitation for optothermal generation and polarization-based phase-shifting for thermo-optic readout (see Fig. 1A, more details in Materials and Methods). Unlike previous mid-IR wide-field optothermal (photothermal) microscopy methods (*14*, *15*, *20*), PSOM suppresses the large intrinsic phase of the sample by means of a unique combination of a polarization phase-shifting mechanism (see Fig. 1, B to F) (*23*) and a MIR-phase retrieve algorithm, thus enhancing chemical contrast sensitivity for live-cell imaging at high cell confluence and for capturing large FOVs. A detailed description of the system is presented in Materials and Methods. Here, briefly, a 532-nm linearly polarized (at 45° in relation to the *x* axis) probe beam is split by a birefringent beam displacer into two orthogonally polarized beams, termed sample beam (SB) and reference beam (RB), which are subsequently rotated 90° by a half-wave plate (see Fig. 1A). The two parallel beams then travel through the sample plane and are recombined by a birefringent beam combiner (Fig. 1B), forming a balanced path Mach-Zehnder interferometer to cancel out the phase noise and reach shot noise limit (*24*). Depending on the optical phase difference between the SB and RB, the resulting recombined beam is either linearly (same phase) or elliptically (different phase) polarized (see Fig. 1, C to E). For the sake of imaging, the recombined beam is collected by a microscope objective with its focus on the sample plane (Fig. 1A). The probe beam then travels through a quarter-wave plate (fast axis at 45°), resulting in a linearly polarized beam with a polarization angle that linearly depends on the optical phase difference between SB and RB (see Fig. 1F). The probe beam keeps its initial polarization angle (α, at 45°) when there is no phase difference between SB and RB and rotates to β when an optical phase difference δ_0_ between SB and RB is introduced (Fig. 1F)—for instance, on SB at the sample plane (Fig. 1B). After passing through an analyzer (i.e., a linear polarizer) and tube lens, the probe beam then forms an image (PSM image) on the camera sensor, where the intensity of the PSM image correlates sinusoidally to the optical phase difference δ_0_ between SB and RB (see figs. S1 to S3). PSM images at different analyzer angles (θ) are acquired to determine the introduced optical phase difference δ_0_ (see section S1).

In PSOM, as can be seen in Fig. 1A, a sample placed in the SB region of the sample plane is illuminated (wide-field illumination; see fig. S4) by a pulsed mid-IR laser beam, at a selected excitation wave number, to generate optical phase perturbation according to molecular vibrational absorption. PSM images are then sequentially acquired at optothermal transition (MIR-ON) and at thermal relaxation between mid-IR laser pulses (MIR-OFF; see Materials and Methods and fig. S5) to generate a subtraction image (MIR-ON minus MIR-OFF; see Fig. 1, G to I). Four subtraction images at four analyzer angles (namely, 315°, 0°, 45°, and 90°) are needed for the phase-shifting mechanism (Fig. 1J). A PSOM micrograph is then obtained by taking the square sum of the subtraction images and applying eq. S11 (see text S2) to generate a phase map (Fig. 1K), which linearly correlates with the sample’s mid-IR absorption at the excitation wave number (see section S3) (*14*). As detailed in section S2, the square sum of the subtraction images suppresses the background from the sample’s intrinsic phase, resulting in enhanced MIR-phase contrast images that exclusively relate to mid-IR absorption.

### System characterization and proof of concept

**Fig. 2.**
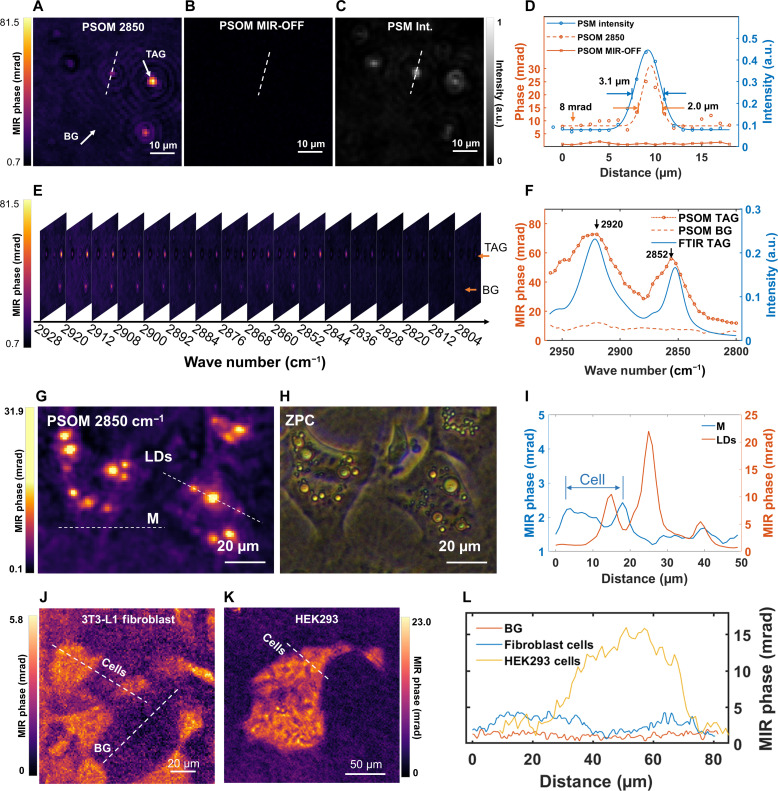
Label-free chemical contrast imaging of synthetic TAG drops and living cells using PSOM. (**A** and **B**) PSOM micrographs of the TAG drops (A) with mid-IR excitation at wave number 2850 cm^−1^ and (B) without mid-IR excitation. BG, background. (**C**) Intensity image acquired by PSM at an analyzer angle of 45°. (**D**) Line profiles crossing the smallest TAG drop in (A) to (C). a.u., arbitrary units. (**E**) PSOM hyperspectral image stack of the same FOV as in (A). (**F**) PSOM spectrum of TAG drop extracted from (E) and its comparison to FTIR measurement. (**G**) PSOM micrograph of single living cells (3T3-L1), demonstrating CH_2_ vibrational contrast obtained at excitation wave number of 2850 cm^−1^. Dotted lines drawn through LDs and plasma membrane (marked as “M”). (**H**) Same FOV as (G) acquired by ZPC microscopy. (**I**) Line profiles crossing LDs and membrane (M) of a cell, as indicated in (G). (**J** and **K**) PSOM image of 3T3-L1 fibroblast cells (J) and a group of HEK293 cells (K) acquired at mid-IR wave number of 2850 cm^−1^. (**L**) Line profiles of the cells from (J) and (K).

First, we tested and characterized the imaging and spectroscopic abilities of PSOM using synthetic TAG phantoms (see Materials and Methods). Figure 2A, for example, depicts a PSOM micrograph of TAG drops in water acquired at a mid-IR excitation wave number of 2850 cm^−1^ (3.509 μm), assigned to symmetric CH_2_ vibration, which generates intrinsic molecular contrast for lipids at a maximum MIR phase of 81.5 mrad. For comparison, Fig. 2B shows a PSOM micrograph of the same FOV with no mid-IR excitation, which results in an image where, as expected, no structures can be observed and from which a spatial noise-equivalent phase of 0.33 mrad can be determined. Furthermore, continuous MIR-OFF measurement of 57 min indicated a temporal noise-equivalent phase of 0.26 mrad (see fig. S6; for calculation of the noise-equivalent phase, see Materials and Methods). Such a low temporal noise level is a quarter of the values reported for state-of-the-art optothermal microscopy (*20*), yielding a contrast-to-noise ratio (CNR; see Materials and Methods) of 282:1 for the marked TAG drop in Fig. 2A. When comparing the imaging resolution of PSOM (Fig. 2A) and PSM (intensity image; Fig. 2C) for the same structure, PSOM image appeared sharper than PSM image despite having been generated by mid-IR excitation, which suggests that PSOM might result in higher imaging resolution compared to PSM. This is demonstrated in Fig. 2D where the intensity profiles across the smallest TAG drop observed in Fig. 2 (A and C) (white dashed line) were compared—the full width at half maximum (FWHM) of the PSOM profile was 2.0 μm, while the FWHM of PSM’s profile was 3.1 μm. The smaller FWHM value from PSOM could have resulted from the fast thermal dissipation around the interface of the TAG drops and water, as noted by other authors using other wide-field optothermal modalities, for instance, BSTP imaging (*14*). For reference, Fig. 2D also shows the MIR-phase profile for the MIR-OFF micrograph in Fig. 2B, where the signal and noise level obtained with PSOM are compared.

Beyond single-wavelength imaging, hyperspectral imaging (Fig. 2E) was achieved for the same FOV in Fig. 2A by acquiring PSOM images while tuning the excitation wave number along a mid-IR spectral range from 2960 to 2800 cm^−1^ in steps of 4 cm^−1^ (for simplicity, only 17 of 41 frames from 2928 to 2804 in steps of 8 cm^−1^ are shown in Fig. 2E). This allowed us to selectively extract the spectra of structures of interest in the FOV, as demonstrated when we plotted the spectra (Fig. 2F) for the structures marked in Fig. 2A (white arrows). Clearly, from Fig. 2F, the spectrum acquired by PSOM showed two absorption peaks characteristic of TAG at 2920 and 2852 cm^−1^, attributed to methylene’s (CH_2_) asymmetric and symmetric stretching vibration, and in good agreement with the spectrum of TAG obtained by conventional FTIR spectroscopy. The MIR phase at 2920 and 2852 cm^−1^ was up to 72.7 and 52.8 mrad, respectively, while, for reference, the spectrum extracted from background (BG), originated from water absorption, remained low at a mean MIR-phase intensity value of 8 mrad. The acquisition time of this hyperspectral imaging stack was 32.8 s (discarding data transferring time, see Materials and Methods for details). Experiments in synthetic TAG phantoms demonstrated a linear response of MIR-phase intensity with mid-IR excitation power (fig. S7), revealing PSOM’s ability to detect TAG drops at an excitation PFD as low as 0.05 μW/μm^2^ at CNR of 26:1. However, to enhance sensitivity in the experiments presented here, we used PFD excitation of 0.16 μW/μm^2^. Our reported PFD is about three times lower than typical values reported for other photothermal imaging modalities (*21*), although it is comparable to values used in ADRIFT (which, however, reports a higher MIR shot noise) (see table S1) (*20*), and up to four orders of magnitude below typical excitation values applied in conventional vibrational imaging modalities such as CARS (15 mW/μm^2^) (*22*).

The exceptionally low mid-IR PFD used in PSOM (0.16 μW/μm^2^) facilitates the application of mid-IR optothermal microscopy to the study of live-cell metabolism by minimizing photodamage exerted on cells (viability test results are shown in fig. S8; viability of HeLa cells was 85.4% after 2 hours of measurement with PSOM). A demonstration of PSOM’s live-cell imaging ability is presented in Fig. 2G for 3T3-L1 differentiated adipocytes, showing lipid contrast obtained at 2850 cm^−1^ (CNR 117:1) generated by LDs formed after 6 days of incubation (see figs. S9 and S10 for corresponding PSM intensity and intrinsic-phase images). The PSOM micrograph in Fig. 2G is comparable to the image obtained by conventional Zernike phase contrast (ZPC) microscopy as shown in Fig. 2H, indicating good morphological correlation between the two techniques. The MIR phase of LDs ranges from around 5 to 22 mrad, while contrast from the phospholipids in the intracellular cell membranes at 2850 cm^−1^ peaks at 2.4 mrad (Fig. 2I). Such a low MIR-phase contrast range (2.4 to 22 mrad) from live-cell structures, at least three orders of magnitude smaller than the intrinsic sample phase (~19 rad difference, from −5.46 to 13.49 rad; see fig. S11H), is difficult to preserve in conventional QPI after application of postprocessing algorithms, which are typically applied to remove phase wrapping (*17**–**19*). Thus, benefiting from intrinsic-phase removal and reduced noise, PSOM is able to detect low-contrast cellular structures rich in CH_2_ bonds, such as cytoplasmic and intracellular membranes. Figure 2 (J and K) shows PSOM images of 3T3-L1 fibroblast cells and human embryonic kidney (HEK) 293 cells, targeting the CH_2_ vibrational signal using a mid-IR excitation wave number of 2850 cm^−1^. Figure 2L shows line intensity profiles crossing the two types of cells. It was observed that fibroblast cells with low confluence (i.e., sparse cell distribution) have MIR phases of around 4 mrad for a single cell. In contrast, highly confluent HEK293 cells, which grow in dense groups, have MIR phases that can reach up to around 15 mrad.

### Implementation of PSOM for large FOV live-cell mid-IR microscopy

**Fig. 3.**
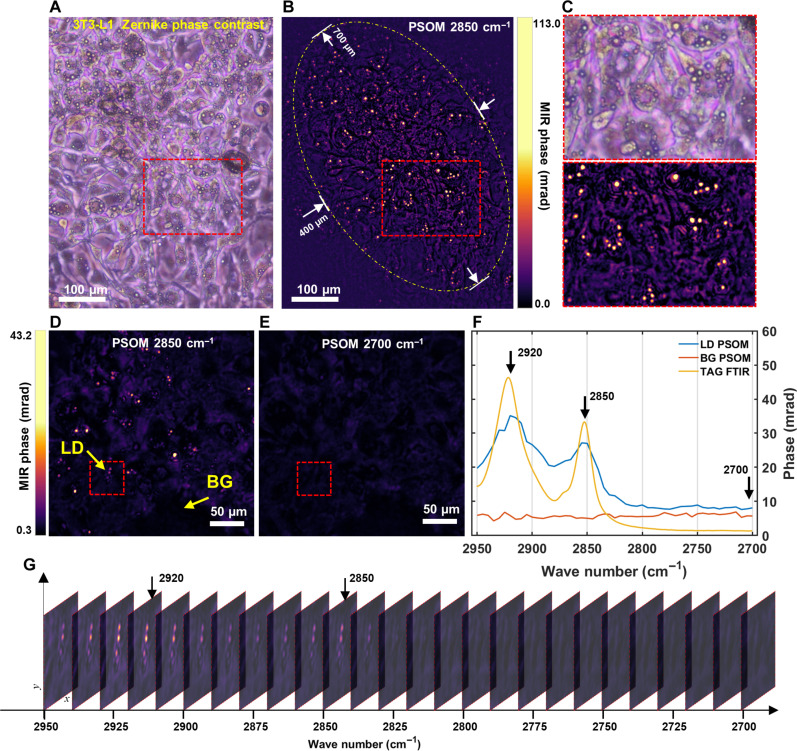
Hyperspectral imaging of highly confluent (100%) living cells at large FOVs by PSOM. (**A**) 3T3-L1 cell micrograph (day 6 after differentiation) obtained by ZPC microscope. (**B**) Chemical contrast micrograph obtained by PSOM for the same FOV as in (A), at wave number 2850 cm^−1^. Dashed ellipse: Effective chemical contrast FOV of 400 × 700 μm. (**C**) Zoomed-in FOVs in red dashed rectangles in (A) and (B). (**D** and **E**) PSOM images of 3T3-L1 cells at (D) CH_2_ resonance wave number 2850 cm^−1^ and (E) off-resonance wave number 2700 cm^−1^. These are two representative images selected from a hyperspectral imaging stack. (**F**) Spectra extracted from hyperspectral imaging stack, showing PSOM spectrum of LD and the spectrum from background (cell media). For comparison, the spectrum of TAG measured by FTIR spectroscopy is plotted. (**G**) Hyperspectral imaging stack for a selected area marked by rectangles in (D) and (E), illustrating chemical contrast change of three LDs from wave number of 2950 to 2700 cm^−1^. LD, lipid droplet; BG, background.

Figure 3 depicts an operational example of large FOV imaging of living cells at high confluence (100%), which has not been demonstrated before by state-of-the-art optothermal microscopy. Measuring highly confluent live cells is important because many cellular processes are only representative of the in vivo state at high confluence, for instance, the differentiation of myoblasts into myotubes and the differentiation of preadipocytes into adipocytes (*25*). Figure 3A shows a ZPC image of 3T3-L1 cells at day 6 after differentiation, displaying an imaging area fully occupied by cells (100% confluence)—for the same FOV, Fig. 3B illustrates a PSOM image at 2850 cm^−1^ with selective lipid contrast. The mid-IR irradiation area for PSOM imaging is an ellipse (major axis: 700 μm, minor axis: 400 μm) as shown by the white dashed line in Fig. 3B, giving an effective chemical contrast FOV of 2.2 × 10^5^ μm. Larger FOVs can be obtained by further expanding the mid-IR excitation area, as shown in fig. S12A for matured 3T3-L1 differentiated cells in a FOV of 5.18 × 10^5^ μm—which is 50 times the demonstrated FOV of state-of-the-art wide-field optothermal microscopy (acquisition time: 0.8 s, discarding data transfer time). Such a large imaging area with living cells at 100% confluence is unprecedented in optothermal microscopy.

Figure 3C shows a zoomed-in FOV of the red dashed area highlighted in Fig. 3 (A and B), used to further compare images from a ZPC microscope and PSOM. In ZPC micrographs, such as in Fig. 3 (A and C), LDs can only be recognized by overall morphology (i.e., with no specific lipid contrast), which makes them hard to distinguish from the large background originating from all molecular content in the cell body—particularly by image segmentation algorithms (see fig. S13). Therefore, quantification of the amount of LDs cannot be easily automated in conventional ZPC micrographs. This confounding background is effectively suppressed in PSOM micrographs (Fig. 3, B and C), where the contrast at 2850 cm^−1^ originates predominantly from the LDs. Unlike measurements at low confluence (such as the single-cell images in Fig. 2G), the MIR phase of LDs in Fig. 3 (B and C) reaches up to 113.0 mrad (CNR 404:1), possibly due to more efficient lipogenesis at 100% confluence as opposed to lower confluence values. Moreover, acquiring the intrinsic phase of highly confluent cell populations by conventional QPI methods is prone to errors due to strong phase wrapping (fig. S11, F and I), which makes conventional QPI-based wide-field optothermal microscopy techniques inadequate for the task of imaging live-cell populations at high confluence. PSOM circumvents this problem by acquiring native MIR-phase images—i.e., independent of intrinsic phase. The capability to image large FOVs enables PSOM to simultaneously capture the heterogeneous activity of cells at different locations and, hence, allows statistical analyses to be carried out on large number of cells to avoid biases that arise from analyzing single cells. As an example, we applied PSOM to determine LD displacement in a large FOV (fig. S12) and LD count (fig. S13), both at high confluency using intrinsic lipid-specific contrast.

Last, Fig. 3 (D and E) shows two PSOM micrographs of 3T3-L1 cells, at a lipid-specific mid-IR wave number of 2850 cm^−1^ and lipid unspecific (off-resonance) wave number of 2700 cm^−1^, used to demonstrate PSOM image specificity to molecular absorption when visualizing living cells. Comparing Fig. 3 (D and E), it can be observed that the LDs only appeared when a wave number of 2850 cm^−1^ was used and were absent when a wave number of 2700 cm^−1^ was used. Moreover, Fig. 3F shows the spectrum of the marked LD in Fig. 3D, as well as the spectrum of a reference point taken from location marked as “BG” in Fig. 3D. The two spectra of LD and BG are plotted together with the TAG spectrum acquired by FTIR. Figure 3G shows a hyperspectral image stack (from mid-IR wave number of 2950 to 2700 cm^−1^ with steps of 5 cm^−1^, only 26 of 51 images are shown for simplicity) of a zoomed-in area as highlighted in Fig. 3 (D and E), illustrating variance of the imaging contrast of LDs over wave numbers. From Fig. 3 (F and G), it can be observed that LDs show high contrast near 2920 and 2850 cm^−1^, and contrast fades out when the mid-IR wave number approaches 2700 cm^−1^. The two absorption peaks can be attributed, respectively, to vibration caused by symmetric and asymmetric stretching of the CH_2_ bond of TAGs, which are the main components in LDs. It is worth noting that the actual image acquisition time for this hyperspectral imaging stack (FOV: 2.5 × 10^5^ μm, corresponding to 2.5 × 10^5^ pixels) with 51 frames was only 41 s (discarding data transferring time), while acquiring similar hyperspectral imaging stacks would take hours for a point-by-point scanning vibrational microscope. For instance, a hyperspectral imaging stack with FOV of 2.25 × 10^4^ pixels took 3.125 hours when using a confocal Raman microscope (*26*).

## DISCUSSION

Here, we presented PSOM for hyperspectral vibrational imaging of highly confluent (up to 100%) living cells in large FOVs. The unique combination of phase-shifting detection and MIR-phase generation achieves suppression of the intrinsic sample phase and enhances sensitivity—allowing the detection of small phase differences induced by vibrational absorption under wide-field mid-IR illumination. Suppression of the intrinsic sample phase does away with the need for postprocessing nonlinear phase unwrapping algorithms. These algorithms are typically required in conventional quantitative phase microscopy for the extraction of MIR phase, and their usage can result in additional imaging artifacts. In particular, with PSOM, we were able to demonstrate snapshot imaging of vibrational contrast of living cells at FOVs up to 5.18 × 10^5^ μm. Besides having a FOV that is approximately 50 times larger than current state of the art (1 × 10^4^ μm; 100 × 100 μm), PSOM achieves this large FOV without computational frame stitching (*21*) and thus avoids the associated disadvantages. Imaging large numbers of cells in large FOVs as opposed to single cells in small FOVs is highly desirable in live-cell microscopy as it allows the achievement of statistical relevance with a small number of quickly and easily obtained snapshots. In addition, benefiting from the intrinsic-phase cancellation brought about by phase-shifting—which yields sub-mrad noise-equivalent phase—using PSOM we were able to measure highly confluent mature adipocytes (containing large organelles) as well as detect the weak vibrational signal (~2.4 mrad) from cell membranes. Moreover, as PSOM is able to provide hyperspectral images from large FOVs, mid-IR spectrum at each pixel in the FOV can be extracted to analyze the distribution of biomolecules of interest in a given cell population—opening up new perspectives for live-cell hyperspectral imaging and its application in label-free metabolic imaging.

As next steps, PSOM could be further developed to achieve faster live-cell hyperspectral imaging speeds by increasing the acquisition imaging frame rate. Frame rate acquisition could be increased, for instance, by (i) using a mid-IR source with a higher repetition rate than the one used here (1 kHz) so that a MIR-ON/OFF imaging cycle can be reduced from 0.5 ms down to 80 μs—physical limit defined by the optothermal cycle (see fig. S14); (ii) implementing a polarization camera to simultaneously acquire images at four different analyzer angles (*27*) (this will reduce the time spent on the mechanical rotation of the analyzer and result in the ability to obtain MIR-phase images in a single snapshot); and (iii) application of onboard image averaging to reduce data-transfer time from camera to computer. In addition, improvements on imaging resolution can be achieved by using a high–numerical aperture (NA) objective instead of the low-NA objective used in our study (NA = 0.28). Furthermore, super-resolution wide-field optothermal imaging can be achieved by analyzing the time-domain optothermal signal (*28*). Last, our current efforts are focused on characterizing and improving PSOM’s sensitivity to absolute absorption coefficients and biomolecular concentration—and although a matter for future publications, in fig. S15, we offer a first glance of PSOM’s sensitivity to optical absorption using water as reference. In water, the lowest absorption coefficient detected was 218 cm^−1^, which generated an MIR phase of 5.4 mrad. Because this value is considerably above the 0.26 mrad of the noise-equivalent phase reported here, we expect PSOM to able to detect even lower absorption coefficients in a future, dedicated, study on sensitivity.

In summary, we foresee PSOM becoming a broadly applied imaging tool in biological research, particularly in live-cell microscopy and single-cell metabolic imaging. For example, as PSOM is a low-irradiation-power label-free molecular imaging modality capable of long-term longitudinal monitoring, unlike fluorescence label–based microscopy, it can continuously capture intrinsic biomolecular contrast over a long time without the risk of photobleaching. Furthermore, beyond the lipid contrast obtained here with an excitation wave number of 2850 cm^−1^ (CH_2_), metabolism of other biomolecules, such as proteins and carbohydrates, could also be studied by extending the spectral coverage to the appropriate fingerprint region, for instance, imaging proteins using the amide bands characterized by absorption peaks between 1700 and 1400 cm^−1^ and imaging carbohydrates that can be detected in the 1300 to 900 cm^−1^ spectral range.

## MATERIALS AND METHODS

### Experimental setup

As shown in Fig. 1A, the probe beam of the PSM was generated by a 532-nm pulse laser (Cobolt 06-Tor, HÜBNER GmbH & Co. KG, 2 kHz), configured to emit a linear polarization beam at an orientation of 90° in relation to the *x* axis. A linear polarizer (LPVISC050-MP2, Thorlabs Inc.) with a transmission axis of 45° was placed in front of the laser to obtain a 45° linearly polarized beam. Next, the linear 45° polarized beam was divided by a birefringent beam displacer (BD27, Thorlabs Inc.) into two orthogonally polarized beams—at 0° and 90° polarization angle with the same intensity. In the beam displacer, the 90° component (termed RB) traveled straight through the beam displacer, while the 0° component (termed SB) deviated 2.7 mm away from the RB path and then continued its travel parallel to RB at the output of beam displacer. The two beams then passed through a half-wave plate (WPHSM05-532, Thorlabs Inc., fast axis: 45°), which rotated the polarization angle of both beams by 90° in relation to the fast axis of the half-wave plate. Therefore, after the half-wave plate, the polarization angle of SB and RB was 90° and 0°, respectively. The two beams then traveled through the sample plane and were, afterward, recombined by a birefringent beam combiner (BD27, Thorlabs Inc.). The combined beam then traveled through the objective (MY10X-803, NA = 0.28; Thorlabs Inc.), which was focused on the sample plane. After the objective, the beam passed through a quarter-wave plate (WPQSM05-532, Thorlabs Inc., fast axis: 45°) and a rotation motor-controlled polarizer (LPVISC050-MP2, Thorlabs Inc., termed analyzer in the main text), for phase-intensity conversion (see section S1). The beam was finally focused by a tube lens to the camera (Dimax cs4, Excelitas PCO GmbH) to form a PSM image.

The pump beam was generated by a mid-IR optical parametric oscillator (OPO) pulse laser (NT277, EKSPLA; 1-kHz pulse repetition rate). A germanium window was used in front of the pump source to filter out the visible “signal” beam generated by the parametric process of OPO. The mid-IR (“idler” beam) was then weakly focused by a low-NA gold parabolic mirror (#37248, Edmund Optics Ltd.; NA = 0.062) on the sample, and the distance of the parabolic mirror to the sample plane was adjusted to achieve the desirable excitation FOV.

The pump and probe laser pulses were synchronized by a trigger pulse generated by a trigger delay generator (Texas Instruments microcontroller, MSP430F5529 LaunchPad). As shown in fig. S5A, the trigger delay generator was connected to a mid-IR laser, visible (Vis) laser, and camera. The mid-IR laser was externally triggered by 1-kHz pulse trains to generate 1-kHz mid-IR pulses (9-ns pulse width), while the Vis laser and camera were externally triggered by 2-kHz pulse trains to generate 2-kHz Vis pulses (2- to 3-ns pulse width) and to capture the image at the time when the Vis pulse arrived at the sample. As shown in fig. S5B, because the repetition rate of the mid-IR pulse was half of the repetition rate of the Vis pulse, half of the images were acquired without mid-IR pulse excitation (MIR-OFF cycle), while the other half of images were acquired with mid-IR pulse excitation (MIR-ON cycle). The time interval between a MIR-ON image and a MIR-OFF image was 0.5 ms. A delay (termed exposure delay) was introduced between mid-IR external trigger and Vis external trigger so that the camera captured the image at exactly the time when the optothermal effect occurred. Varying this exposure delay results in the time-dependent optothermal transient signal as shown in figs. S14 and S16. In living cell measurements, we averaged 200 PSOM images to increase the CNR, which required 1600 PSM images in total for a final PSOM image (eight PSM images were required for a single PSOM image, as shown in Fig. 1, G to J), acquired from Vis using 1600 trigger pulses within 0.8 s.

### Preparation of 3T3-L1 white adipocytes

A mouse fibroblast cell line (3T3-L1, American Type Culture Collection: CRL-1658) was used as an in vitro model of white adipocytes. The preadipocytes were plated in custom-made plates (fig. S17) and cultured in growth medium composed of low-glucose (1 g liter^−1^) Dulbecco’s modified Eagle’s medium (DMEM; Merck) supplemented with 10% fetal bovine serum (FBS; Merck) and 1% penicillin-streptomycin (Merck). Once 100% confluence was reached, the cells were differentiated to adipocytes. For this purpose, a differentiation medium, composed of high-glucose (4.5 g liter^−1^) DMEM (Merck), 10% FBS, 1% penicillin-streptomycin, insulin (1 μg ml^−1^; Sigma-Aldrich), 0.25 μM dexamethasone (Sigma-Aldrich), 0.5 mM 3-isobutyl-1-methylxanthine (Sigma-Aldrich), and 1:1000 volume ABP [l-ascorbate (50 mg ml^−1^), 1 mM biotin, 17 mM pantothenate (Sigma-Aldrich)], was added to the cells on days 0 and 2. On day 4, the cells were cultured with differentiation medium supplemented with insulin (1 μg ml^−1^) and 1:1000 volume ABP. On day 6, the differentiation medium was changed back to growth medium (low glucose). Cells were kept in an incubator at 37°C with 5% CO_2_ and measured by PSOM in growth medium (low glucose). A cell-free region for the RB was created by covering half of the dish with a medical-grade tape (ARcare 90445Q, Adhesives Research Inc.), which was removed during PSOM measurement.

### Preparation of TAG drops

A suspension (10 mg/ml) of TAG drops was prepared by dissolving 1 mg of 1,2-dioleoyl-3-palmitoyl-rac-glycerol (Sigma-Aldrich Inc.) in 100 μl of a chloroform-methanol solution (2:1). Ten microliters of the TAG drop suspension was pipetted on a custom-made dish (fig. S17; with a ZnS window as dish bottom) and left to dry at room temperature until the chloroform and methanol were completely evaporated, leaving the TAG drops on the surface of the ZnS window. After evaporation of chloroform and methanol, the dish was perfused with deionized water and covered with a cover glass, maintaining a 3-mm depth of water in the dish during the measurement. On the ZnS window, a reference region was maintained without any TAG drops for the RB.

### Viability test

For the viability test, we plated HeLa cells in six custom-made dishes and cultured the cells until 80% confluence was reached. The cells were divided into two groups: Dishes 1 to 3 were measured by PSOM, and dishes 4 to 6 were used as controls. Dishes 1 to 3 were measured continuously for 2 hours on the PSOM stage, with one dish used per wave number tested (2850, 1550, or 1100 cm^−1^). At these three wave numbers, mid-IR PFD on a sample was 0.165, 0.027, and 0.024 μW/μm^2^, respectively. The corresponding control dishes (dishes 4 to 6) were maintained in the same condition (room temperature) without mid-IR illumination. Immediately after finishing the test, to assess the negligibility of photodamage induced by PSOM, standard erythrosine B exclusion assays were performed. The cells in all the dishes (control and test) were stained with erythrosine B before counting them, and cell viability was expressed as the percentage ratio of viable irradiated cells compared to the corresponding viable non-irradiated controls. For determination of statistical significance, OriginPro9.1 software was used. Reported data corresponded to the mean ± SD from three measurements.

### CNR and noise level

The CNR, which takes the value of 0.26 mrad according to fig. S6, is defined as the MIR-phase difference between the signal and the background (φ_signal_ − φ_BG_) divided by the temporal noise-equivalent phase σ*_s_*, as follows

CNR=φsignal−φBGσs (1)

where temporal noise-equivalent phase σ*_s_* is calculated from 57 min of a PSOM video (fig. S6) without mid-IR excitation, using

$$\sigma_{s}=\sqrt{\frac{1}{5N}\sum_{i}^{5N}\left(\varphi_{i}-\mu \right)}$$ (2)

φ*_i_* is the MIR phase at pixel index *i* from the MIR-OFF PSOM video, and μ is the average of MIR phase among 5*N* pixels, *N* is the number of frames in the video, and pixels are chosen from five locations as shown in fig. S6A. For calculating spatial noise-equivalent phase, all pixels of a single frame are used.

### Data processing

PSOM images were processed by ImageJ. Illustration of spectra and profiles was achieved by MATLAB. For images shown in Fig. 2 (G, J, and K), the water absorption background (determined in cell-free region) was subtracted from the overall image, for better illustration of the cell contrast. The tracing of LDs in figs. S12 and S13 was achieved by TrackMate.

## Supplementary Materials

### This PDF file includes:

Sections S1 to S6 Figs. S1 to S17 Tables S1 and S2 References

Correction (19 April 2024):
Due to a production error, the incorrect reference list was attached to the end of the Supplementary Materials PDF. The PDF has been updated to include the correct reference list. The original version is available here:
