# Supplementary material for: Phase-shifting optothermal microscopy enables live-cell mid-infrared hyperspectral imaging of large cell populations at high confluency
Source: Sci Adv. 2024 Feb 21;10(8):eadj7944. doi: 10.1126/sciadv.adj7944 (PMC10881023; doi:10.1126/sciadv.adj7944)
Supplement: Supplementary file 1 — Sections S1 to S6 Figs. S1 to S17 Tables S1 and S2 References [file sciadv.adj7944_sm.v2.pdf]

CORRECTED 19 APRIL 2024; SEE FULL TEXT

Science Advances
AAAS

Supplementary Materials for

Phase-shifting optothermal microscopy enables live-cell mid-infrared hyperspectral imaging of large cell populations at high confluency

Tao Yuan *et al.*

Corresponding author: Miguel A. Pleitez, miguel.pleitez@tum.de

Sci. Adv. **10**, eadj7944 (2024)
DOI: 10.1126/sciadv.adj7944

This PDF file includes:

Sections S1 to S6
Figs. S1 to S17
Tables S1 and S2
References

Supplementary Materials

1. Phase-Intensity relationship

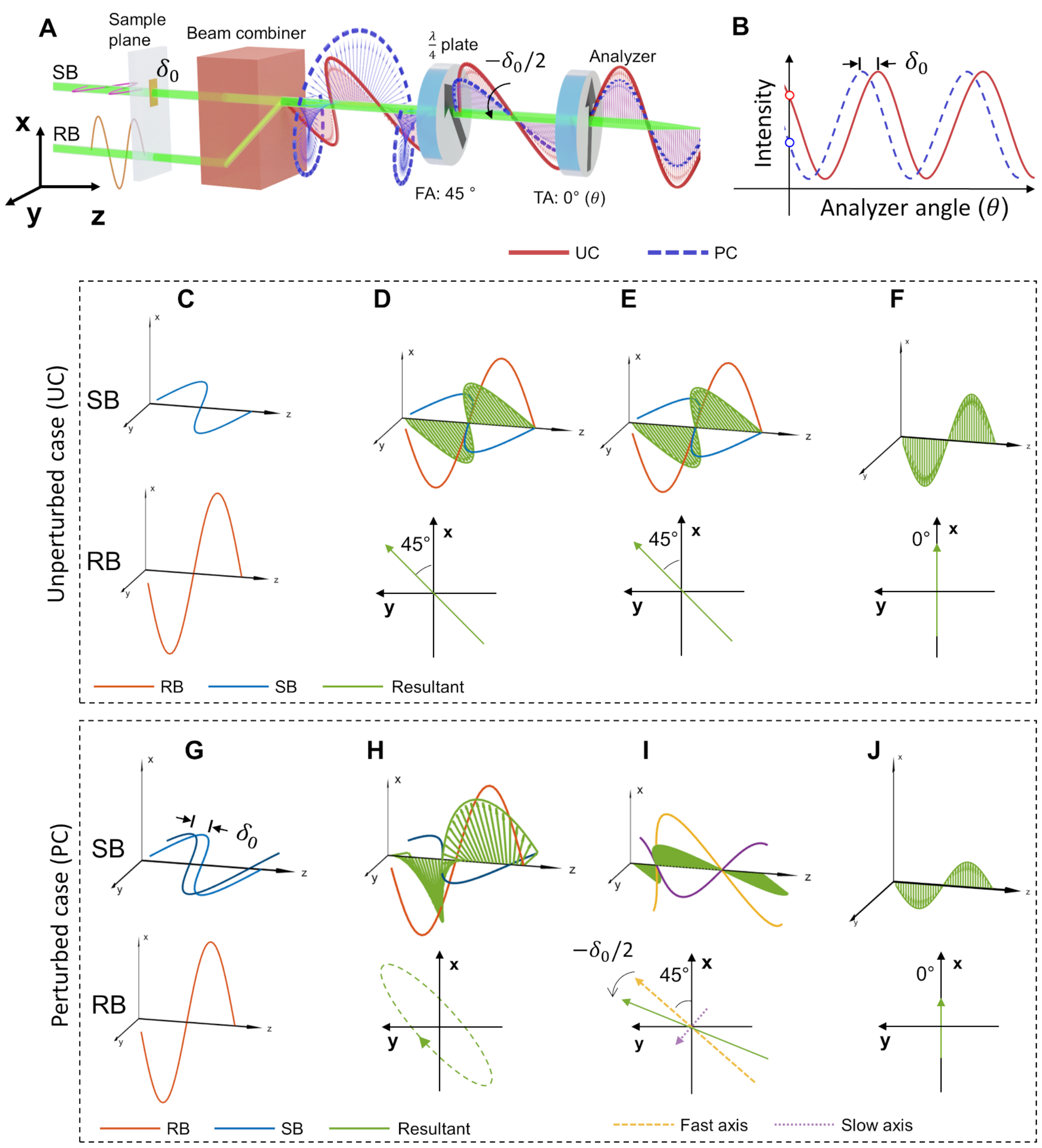

Fig. S1. Polarization states of the probe beam as it travels through the optics. A, 3D diagram of the system emphasizing the polarization states of the probe beam in the unperturbed case (UC) and the perturbed case (PC). **B,** Plot of output intensity varying with analyzer angle. **C-F,** Polarization states of probe beam after (C) sample plane, (D) beam combiner, (E) quarter-wave plate, and (F) analyzer, in the unperturbed case. **G-J**, Polarization states of probe beam after (G) sample plane, (H) beam combiner, (I) quarter-wave plate, and (J) analyzer, in the perturbed case. Second row of (D-F) and (H-J) illustrate the polarization in x-y plane. SB: Sample beam; RB: Reference beam. FA: Fast axis; TA: Transmission Axis.

Fig. S1A,B illustrates the polarization states of the probe-beam when interacting with the optics of the system (**Fig. S1A**), and a plot of output intensity varying with analyzer angle (**Fig. S1B**). When both beams, sample beam (SB) and reference beam (RB), keep the same optical phase (i.e., unperturbed case**,** see **Fig. S1C**) the combined probe beam out of the beam combiner is a 45° linearly polarized beam (**Fig. S1D**). After passing through a quarter-wave plate (fast axis: 45°, **Fig. S1A**), the combined beam keeps its 45° linear polarization state (**Fig. S1E**). If the analyzer (polarization angle can be controlled) is at 0°, only the x-axis polarization component can transmitted thought the analyzer (**Fig. S1F**), corresponding to intensity value marked by the red circle in **Fig. S1B**.

When an optical perturbation δ_0 (can be the intrinsic-phase or MIR-phase) is introduced to the SB on the sample plane (i.e., perturbed case**,** see **Fig. S1G**), after the beam combiner, an elliptically polarized beam is the result (**Fig. S1H**). After the quarter-wave plate, this elliptical polarization beam is converted back to a linearly polarized beam (**Fig. S1I**). As shown in **Fig. S1I**, the polarization plane of the linearly polarized beam (green sinusoid wave, or green sinusoid arrow) has an angle $-\delta_0/2$ rotated relative to the unperturbed case (45°), where the minus sign stems from the fact that negative phase change (phase retardation) results in positive rotation (counterclockwise rotation). As with the unperturbed case discussed above, the analyzer (transmission angle: 0°) only allows the x-axis component to pass through (**Fig. S1J**), and in this case corresponds to a lower intensity value marked by the blue circle in **Fig. S1B**. The output intensity after the analyzer follows Malus' law, which states that the intensity (I) of light that passes through an analyzer varies with the angle (θ_i) between the light's polarization plane and the transmission axis of the analyzer, following the relationship: $I = I_0 cos^2\theta_i$, where I_0 is the light intensity before entering the analyzer. Using a defined angular coordinate (defined as the counterclockwise rotation angle from positive semi-axis of x), the angle θ_i can be written as $\theta_i = \pi/4 - \delta_0/2 - \theta$, where θ is the analyzer angle. When θ_i in Malus' law is substituted by this expression, we have a phase-intensity relationship:

$$I = \frac{I_0}{2}\left(1 + sin(2\theta + \delta_0)\right) \qquad eq.S1$$

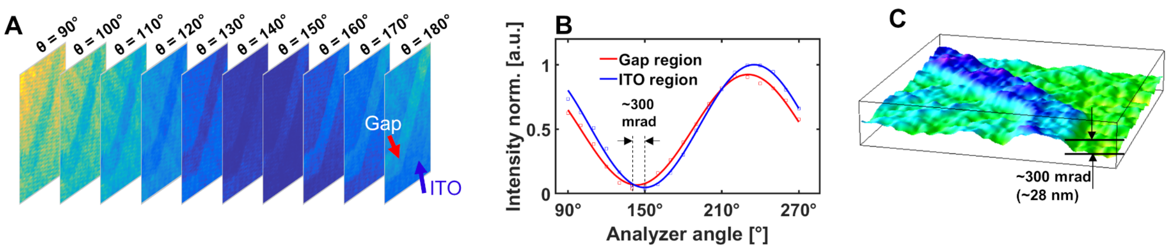

Fig. S2. Validation of phase-intensity sinusoid relationship using an indium tin oxide (ITO) phase target. A, Intensity image stack acquired by adjusting the angle of the analyzer (θ) from 90° to 180° with steps of 10°. Red arrow: a point on the Gap area (with absent ITO layer); Blue arrow: a point on the ITO layer. **B,** The two points chosen from the image stack were fitted to a sinusoidal function, and the phase difference between Gap and ITO pints can be obtained as indicated. **C,** Quantitative phase image of ITO phase target constructed from the phase difference obtained at each location of the whole image plane.

2. Cancellation of intrinsic phase

For MIR-OFF images, the phase perturbation is caused by refractive index and thickness of the sample (i.e., intrinsic-phase) denoted here by $\phi_{0(x,y)}$, where x, y indicates the coordinates of the local phase perturbation in the sample plane. Following **eq. S1**, a phase-shifting module (PSM) intensity image with MIR-OFF can be written as: $I_{f(x,y)} = I_0/2\left(1 + sin\left(2\theta + \phi_{0(x,y)}\right)\right)$. When the sample is irradiated by mid-IR light (generated by pump laser as shown in **Fig. 1A** in the main text), absorption of mid-IR light by the sample creates a localized temperature change $T_{(x,y)}$, hence a local phase perturbation $\Delta\varphi_{(x,y)}$ (i.e. MIR-phase). As a result, the total phase perturbation is the sum of intrinsic-phase $\phi_{0(x,y)}$ and MIR-phase $\Delta\varphi_{(x,y)}$. Following **eq. S1**, an intensity image with MIR-ON can be written as: $I_{n(x,y)} = I_0/2\left(1 + sin\left(2\theta + \phi_{0(x,y)} + \Delta\varphi_{(x,y)}\right)\right)$. One can make a subtraction to obtain the intensity difference between MIR-ON and MIR-OFF images: $\Delta I_{(x,y)} = I_{n(x,y)} - I_{f(x,y)}$.

In PSOM, the phase-shifting mechanism requires four intensity subtraction images: $\Delta I_{1(x,y)}$, $\Delta I_{2(x,y)}$, $\Delta I_{3(x,y)}$, $\Delta I_{4(x,y)}$ from analyzer angles 315°, 0°, 45° and 90° (see **Fig. 1J** in main text). According to the phase-intensity relationship given in **eq. S1**, the four subtraction images from 4 analyzer angles (315°, 0°, 45°, or 90°) can be calculated as (after normalizing the initial intensity I_0 to 1, and substituting the values of θ):

$$\Delta I_{1(x,y)} = sin\left(\phi_{0(x,y)} + \frac{\Delta\varphi_{(x,y)}}{2}\right) sin\left(\frac{\Delta\varphi_{(x,y)}}{2}\right) \qquad eq.\mathrm{S2}$$

$$\Delta I_{2(\mathrm{x,y})} = cos\left(\phi_{0(x,y)} + \frac{\Delta\varphi_{(x,y)}}{2}\right) sin\left(\frac{\Delta\varphi_{(x,y)}}{2}\right) \qquad eq.\mathrm{S3}$$

$$\Delta I_{3(\mathrm{x,y})} = -sin\left(\phi_{0(x,y)} + \frac{\Delta\varphi_{(x,y)}}{2}\right) sin\left(\frac{\Delta\varphi_{(x,y)}}{2}\right) \qquad eq.\mathrm{S4}$$

$$\Delta I_{4(\mathrm{x,y})} = -cos\left(\phi_{0(x,y)} + \frac{\Delta\varphi_{(x,y)}}{2}\right) sin\left(\frac{\Delta\varphi_{(x,y)}}{2}\right) \qquad eq.\mathrm{S5}$$

As can be seen above, the subtraction image is expressed as the multiplication of two parts: the sine or cosine function of the sum $\phi_{0(x,y)} + \frac{\Delta\varphi_{(x,y)}}{2}$, and a common term $sin\left(\frac{\Delta\varphi_{(x,y)}}{2}\right)$ shared by **eq. S2-5**. The former is dependent on intrinsic-phase $\phi_{0(x,y)}$, while the latter is independent of intrinsic-phase $\phi_{0(x,y)}$. Following the procedure described in **Fig. 1J,K**, we take the square sum of these 4 subtraction images:

$$\sigma_{(x,y)}{}^2 = \Delta I_{1(x,y)}{}^2 + \Delta I_{2(x,y)}{}^2 + \Delta I_{3(x,y)}{}^2 + \Delta I_{4(x,y)}{}^2 \qquad eq.\mathrm{S6}$$

This results in:

$$\sigma_{(x,y)}{}^2 = 2\left[sin^2\left(\phi_{0(x,y)} + \frac{\Delta\varphi_{(x,y)}}{2}\right) + cos^2\left(\phi_{0(x,y)} + \frac{\Delta\varphi_{(x,y)}}{2}\right)\right] sin^2\left(\frac{\Delta\varphi_{(x,y)}}{2}\right) \qquad eq.S7$$

Using the formula: $sin^2(\alpha) + cos^2(\alpha) = 1$, one can easily cancel out the dependence of the intrinsic-phase $\phi_{0(x,y)}$ and obtain:

$$\sigma^2{}_{(x,y)} = 2sin^2\left(\frac{\Delta\varphi_{(x,y)}}{2}\right) \qquad eq.S8$$

Next, we calculate the square root of both sides:

$$\sigma_{(x,y)} = \sqrt{2}\left|sin\frac{\Delta\varphi_{(x,y)}}{2}\right| \qquad eq.S9$$

Because of the cancellation carried out in **eq. S7**, the resulting $\sigma_{(x,y)}$ in **eq. S9** is independent of the intrinsic-phase $\phi_{0(x,y)}$, which is several orders of magnitude larger than the MIR-phase. The intrinsic-phase $\phi_{0(\mathrm{x,y})}$ of a cell can be more than 2π rad (*31, 32*) which leads to phase wrapping artifact. In the phase wrapping artifact, all the phase ranges [2$k\pi$, 2$k\pi$ +2π) are mapped to the range [0, 2π) (where, k = …-2,-1,0,1,2…). In the mapping relationship, the mapped phase wraps back to 0 every time the actual phase exceeds 2$k\pi$. The phase unwrapping algorithms are usually used for recovering actual phase images, but they are error-prone and may introduce errors larger than the MIR-phase. This susceptibility to errors is even more of a challenge when dealing with images from large FOVs, where the spatial phase wrapping occurs more frequently than the pixel step of the camera. The unique feature of PSOM is that it can remove the intrinsic-phase without obtaining it, circumventing the need for phase unwrapping to recover the intrinsic-phase, and making optothermal imaging of large FOVs possible. When the considered MIR-phase shift $\Delta\varphi_{(x,y)}$ is very small, we can make the following approximation from **eq. S9**:

$$\sigma_{(x,y)} \approx \left|\frac{\sqrt{2}\Delta\varphi_{(x,y)}}{2}\right| \qquad eq.S10$$

Note that the approximation can be made when the intensity images of MIR-ON/OFF are normalized to [1,0]. **Eq. S10** states that the absolute quantitative MIR-phase $\varphi_{(x,y)}$ can be obtained by multiplying $\sigma_{(x,y)}$ by a factor ($\sqrt{2}$) for the construction of a PSOM image:

$$\left|\Delta\varphi_{(x,y)}\right| \approx \sqrt{2}\sigma_{(x,y)} \qquad eq.S11$$

3. Relationship between the mid-IR absorption coefficient and MIR-phase

The relationship between the vibrational absorption coefficient and MIR-phase $\Delta\varphi$ of a PSOM image is linearly correlated according to ref. (*14*) as follow:

$$\frac{2\pi E}{\lambda A}\frac{l(\alpha + n\beta)}{c\rho}\mu_{\omega} = \Delta\varphi \qquad eq.S12$$

Here, μ_{ω} is the absorption coefficient at wavelength ω, E is the energy of a single mid-IR pulse, λ is the wavelength of visible (VIS) probe beam, A is irradiation area of the mid-IR light and the term $\frac{2\pi E}{\lambda A}$ is system dependent. In contrast, the term $\frac{l(\alpha+n\beta)}{c\rho}$ is sample dependent, where c is specific heat capacity of sample, ρ is density of the sample, l is the thickness of the sample, n is the refractive index of the sample, $\alpha = \frac{dn}{dT}$ is the thermo-optic coefficient of the sample and $\beta = \frac{1}{l}\frac{dl}{dT}$ is the linear thermal expansion coefficient.
For a given sample measured in a given system, $\frac{2\pi E}{\lambda A}\frac{l(\alpha+n\beta)}{c\rho}$ is a constant, and eq. S12 can be written as a linear relationship: $\kappa c\mu_{\omega} = \Delta\varphi$.

In summary, the MIR-phase is wavelength dependent, and the mid-IR absorption spectrum can be extracted from the PSOM image stack, which is acquired under different mid-IR wavelength.

4. Quantitative phase imaging with PSM

According to the phase-intensity formula (**eq. S1**), 4 frames of MIR-OFF intensity images at analyzer angles of 315°, 0°, 45° and 90° can be expressed as:

$$I_{1(x,y)} = I_0/2\left(1 + sin\left(630° + \phi_{0(x,y)}\right)\right) \qquad eq.S13$$

$$I_{2(x,y)} = I_0/2\left(1 + sin\left(0° + \phi_{0(x,y)}\right)\right) \qquad eq.S14$$

$$I_{3(x,y)} = I_0/2\left(1 + sin\left(90° + \phi_{0(x,y)}\right)\right) \qquad eq.S15$$

$$I_{4(x,y)} = I_0/2\left(1 + sin\left(180° + \phi_{0(x,y)}\right)\right) \qquad eq.S16$$

The tangent of the intrinsic-phase can be derived using the above 4 PSM intensity images.

$$\frac{I_{2(x,y)} - I_{4(x,y)}}{I_{3(x,y)} - I_{1(x,y)}} = \frac{I_0/2\left(1 + sin\left(\phi_{0(x,y)}\right) - 1 + sin\left(\phi_{0(x,y)}\right)\right)}{I_0/2\left(1 + cos\left(\phi_{0(x,y)}\right) - 1 + cos\left(\phi_{0(x,y)}\right)\right)} = \frac{sin\left(\phi_{0(x,y)}\right)}{cos\left(\phi_{0(x,y)}\right)} = tan\left(\phi_{0(x,y)}\right) \qquad eq.S17$$

Therefore, we can obtain the quantitative phase image from these 4 PSM intensity images:

$$\phi_{0(x,y)} = tan^{-1}\left(\frac{I_{2(x,y)} - I_{4(x,y)}}{I_{3(x,y)} - I_{1(x,y)}}\right) \qquad eq.S18$$

5. Jones vectors at each position of the setup

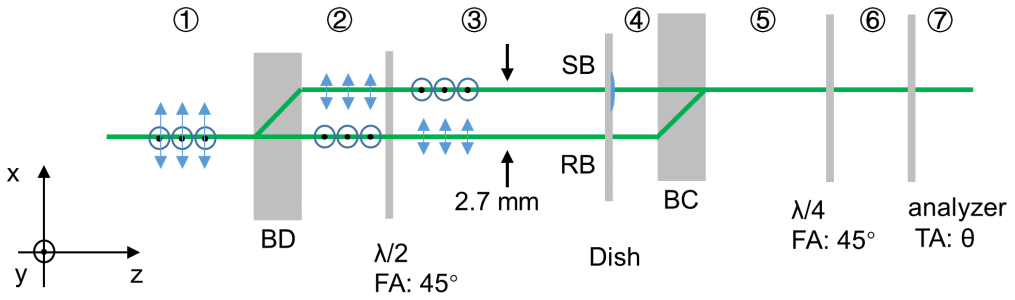

Fig. S3. Diagram of the polarization phase-shifting interferometer in PSOM. The polarization phase-shifting interferometer uses a 45° linear polarized light as input. A birefringent crystal (beam displacer, BD) divides the beam into two paths with orthogonal polarization directions: the sample beam (SB) with x-axis polarization, and the reference beam (RB) with y-axis polarization. Afterward, the two beams travel through a half-wave plate with fast axis of 45°, which rotates the polarization of both beams by 90°. Then the sample beam travels through the sample so that a phase difference between the sample beam and the reference beam is introduced. The two beams then travel through a birefringent beam combiner (BC) to be combined. Finally, the combined beam travels through a quarter wave plate and an analyzer. FA: Fast Axis; TA: Transmission Axis.

In **Fig. S3**, assuming the amplitude of the input beam I_0 as unit 1, the Jones vector in position ① is:

$$\frac{1}{\sqrt{2}}\begin{pmatrix}1\\1\end{pmatrix} \qquad eq.S19$$

The Jones vectors in position ② are:

(i) Reference beam:

$$\frac{1}{\sqrt{2}}\begin{pmatrix}0\\1\end{pmatrix} \qquad eq.S20$$

(ii) Sample beam:

$$\frac{1}{\sqrt{2}}\begin{pmatrix}1\\0\end{pmatrix} \qquad eq.S21$$

In position ③, due to the half-wave plate, the Jones vectors become:

(i) Reference beam:

$$\frac{1}{\sqrt{2}}e^{-i\frac{\pi}{2}}\begin{pmatrix}1\\0\end{pmatrix} \qquad eq.S22$$

(ii) Sample beam:

$$\frac{1}{\sqrt{2}} e^{-i\frac{\pi}{2}} \begin{pmatrix} 0 \\ 1 \end{pmatrix} \qquad eq.S23$$

In position ④, we assume that a phase difference δ is introduced to the sample beam, therefore the Jones vector of the sample beam becomes:

$$\frac{1}{\sqrt{2}} e^{-i\frac{\pi}{2}} e^{i\delta} \begin{pmatrix} 0 \\ 1 \end{pmatrix} \qquad eq.S24$$

The reference beam in position ④ keeps the same Jones vector as before the sample plane:

$$\frac{1}{\sqrt{2}} e^{-i\frac{\pi}{2}} \begin{pmatrix} 1 \\ 0 \end{pmatrix} \qquad eq.S25$$

In position ⑤, the two beams are combined, with the Jones vector of:

$$\frac{1}{\sqrt{2}} e^{-i\frac{\pi}{2}} \begin{pmatrix} 1 \\ e^{i\delta} \end{pmatrix} \qquad eq.S26$$

In location ⑥, since the Jones matrix for the 45° quarter wave plate (QWP) is: $\frac{1}{2} e^{-i\frac{\pi}{4}} \begin{pmatrix} 1+i & 1-i \\ 1-i & 1+i \end{pmatrix}$, the Jones vector of the combined beam is:

$$\frac{1}{2\sqrt{2}} e^{-i\frac{\pi}{4}} e^{-i\frac{\pi}{2}} \begin{pmatrix} 1+i+(1-i)e^{i\delta} \\ 1-i+(1+i)e^{i\delta} \end{pmatrix} \qquad eq.S27$$

In location ⑦, the Jones matrix for the linear polarizer (i.e., analyzer) with the transmission axis angle θ can be expressed as:

$$\begin{pmatrix} cos^2\theta & sin\theta cos\theta \\ sin\theta cos\theta & sin^2\theta \end{pmatrix} \qquad eq.S28$$

The Jones vector in position ⑦ is:

$$\begin{pmatrix} cos^2\theta & sin\theta cos\theta \\ sin\theta cos\theta & sin^2\theta \end{pmatrix} \cdot \frac{1}{2\sqrt{2}} e^{-i\frac{\pi}{4}} e^{-i\frac{\pi}{2}} \begin{pmatrix} 1+i+(1-i)e^{i\delta} \\ 1-i+(1+i)e^{i\delta} \end{pmatrix} \qquad eq.S29$$

Since the Jones vector represents the amplitude and phase of the electric field, using the Jones vector in ⑦ , one can calculate the output intensity after the analyzer:

$$I = \frac{1}{2}\big(1 + sin(2\theta + \delta)\big) \qquad eq.S30$$

where θ is the polarization angle, and δ is the phase difference introduced by the sample. Eq. S30 states the relationship between the intensity of the interferogram (I), the analyzer angle (θ), and the phase

retardation introduced by the sample (δ). This relationship is consistent with relationship given in eq. S1 (here we assume the initial light intensity as 1).

6. Figures

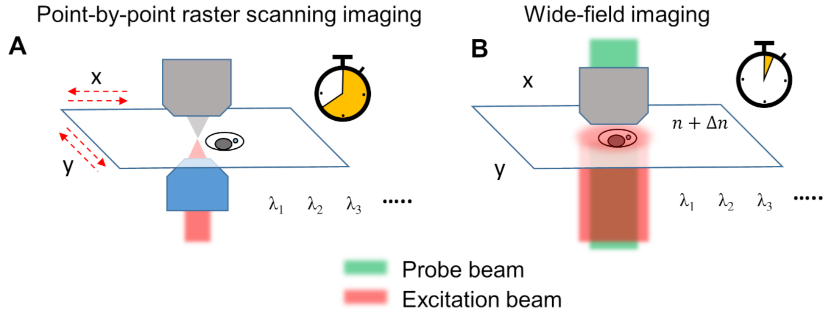

Fig. S4. Comparison of point-by-point raster scanning imaging and wide-field imaging. A, In point-by-point scanning imaging, the excitation beam is focused into a diffraction limit spot, and an image is constructed from data acquired at each point in the field-of-view (FOV). Due to the scanning mechanism, hyperspectral imaging from multiple excitation wavelengths ($\lambda_1, \lambda_2, \lambda_3 \ldots$) proceeds slowly. **B,** In wide-field imaging, a broad mid-IR beam is used for excitation. Visualization of a sample in a large FOV can be achieved quickly via capturing a snapshot of detected optothermal-related phase change.

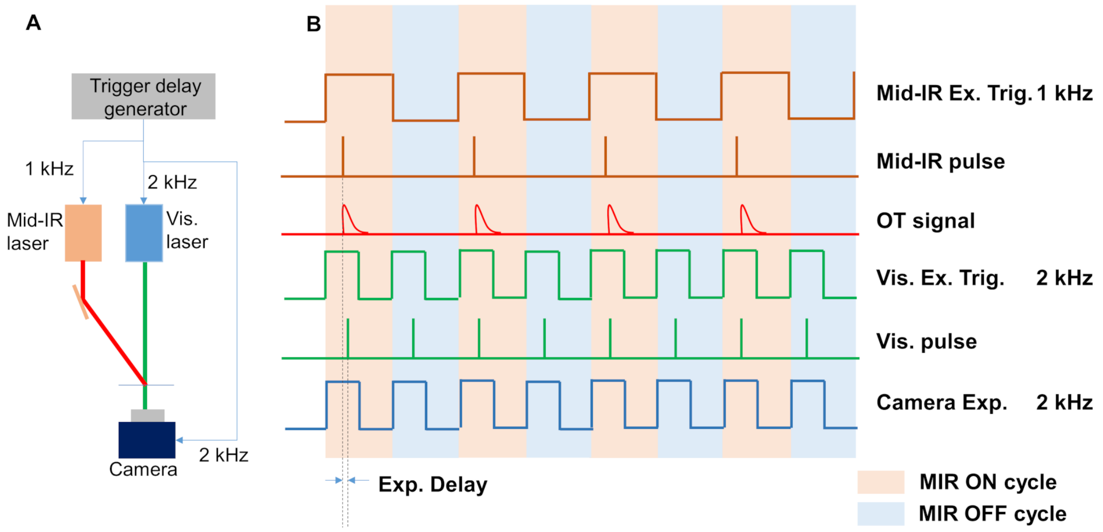

Fig. S5. System synchronization. **A**, Simplified diagram of PSOM, emphasizing the synchronization connection and pump-probe mechanism. **B,** Diagram of the trigger pulse trains and their corresponding mid-IR/Vis. pulse outputs, and optothermal (OT) signals. Mid-IR Ex. Trig.: External trigger for the mid-infrared optical parametric oscillator (OPO). Vis. Ex. Trig.: External trigger for the 532 nm pulse laser. Camera Exp.: External trigger for camera exposure. The three synchronized trigger signals (mid-IR Ex. Trig., Vis. Ex. Trig. and Camera Exp.) are generated by a trigger delay generator, where the mid-IR Ex. Trig. is a 1 kHz square pulse train, both Vis. Ex. Trig. and Camera Exp. are 2 kHz square pulse trains. As demonstrated in (B), the camera operates with a frame rate of 2 kHz, triggered by the 2 kHz signal; the exposure cycle with mid-IR pulse is called the MIR ON cycle, while the cycle without mid-IR pulse is called the MIR OFF cycle, the corresponding images are referred to as MIR-ON images and MIR-OFF images, respectively. An exposure delay (Exp. Delay) is strategically introduced to the two 2 kHz signals (Vis. Ex. Trig. and Camera Exp.),

so that the mid-IR pulse and Vis. pulse reach the sample at the same time. Time-dependent optothermal transient signal can be obtained by varying the exposure delay.

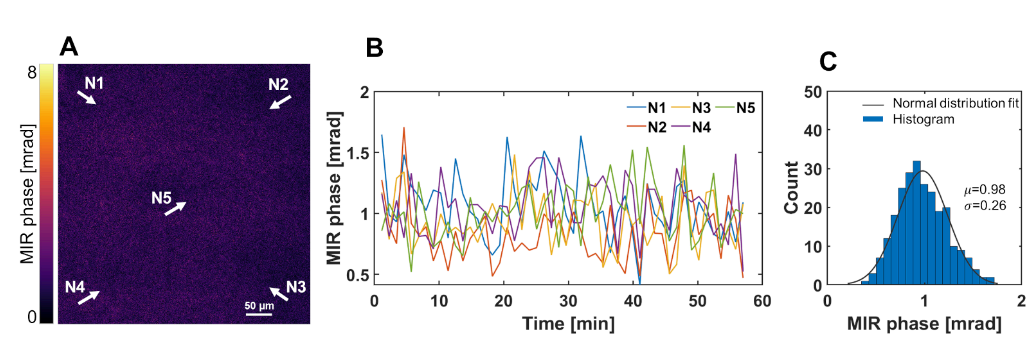

Fig. S6. Noise measurement over time. A, A PSOM image acquired without mid-IR irradiating the FOV. **B,** Temporal change of MIR-phase from 5 locations of the FOV, as marked in (A). Measurement duration: 57 minutes. **C,** Histogram of all the temporal data points from (B), and the corresponding normal distribution fit curve. μ: mean; σ: standard deviation. Temporal noise-equivalent phase of 0.26 mrad is obtained (see **Materials & Methods**), corresponding to an equivalent optical path length of 22 pm (considering visible laser wavelength of 532 nm).

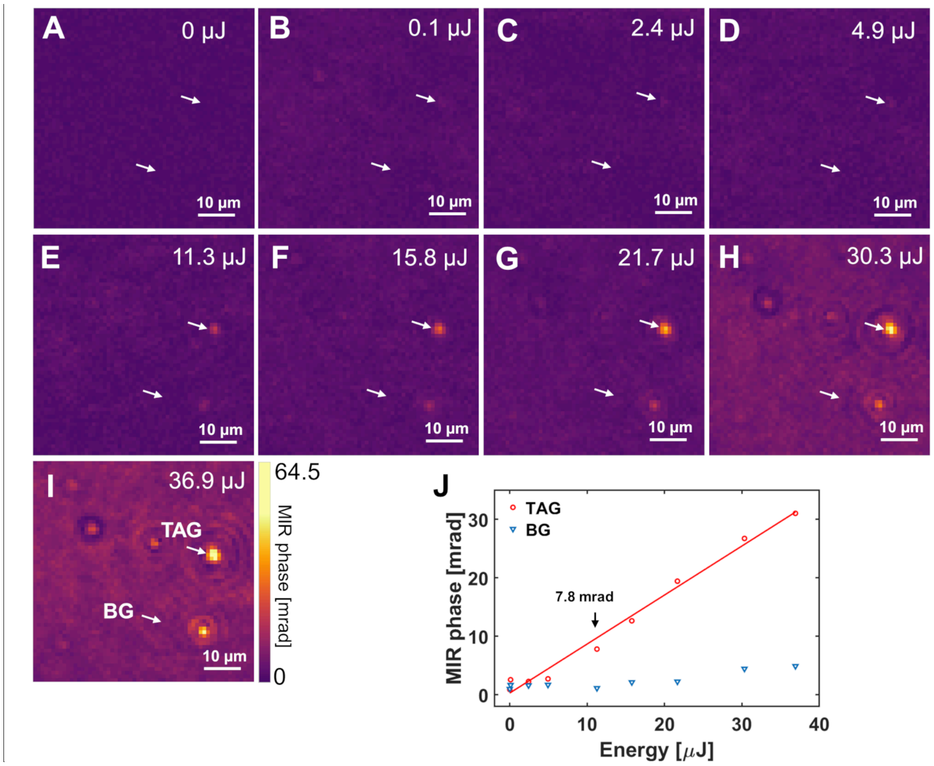

Fig. S7. Linear relationship between mid-IR pulse energy and MIR-phase of a PSOM image. A-I, PSOM images with different mid-IR pulse energy directed on the sample, ranging from 0 μJ to 36.9 μJ. The visibility of synthetic TAG drops increases as the pulse energy increases from (A) to (I). **J,** Plot of the MIR-phase values at a synthetic TAG drop and in an area selected for acquiring background (BG) values. The MIR-phase of a particular TAG drop, indicated in (I), is linearly correlated with excitation energy. In (E), under mid-IR pulse energy of 11.3 μJ/pulse (corresponding to power flux density of 0.05 μW/μm^2), the TAG drop can be distinguished with a CNR of 26:1 (MIR-phase of 7.8 mrad).

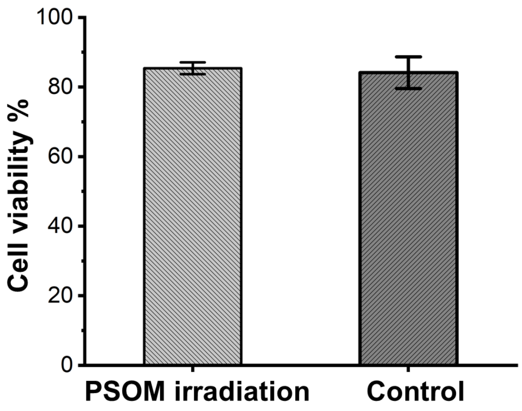

Fig. S8. Viability assessment of HeLa cells imaged with PSOM. In this test HeLa cells were imaged continuously for 2 h with PSOM. The test was performed three times on three different dishes for each condition, with or without mid-IR irradiation, (N=3 for each condition). The controls were prepared and maintained in the same condition (room temperature) as the irradiated samples, but without mid-IR excitation. The bars represent the mean cell viability of PSOM irradiated (85.4%) samples and control (83.8%). The error bars represent the standard deviation (PSOM irradiated samples: 3.9%; control: 8.2%).

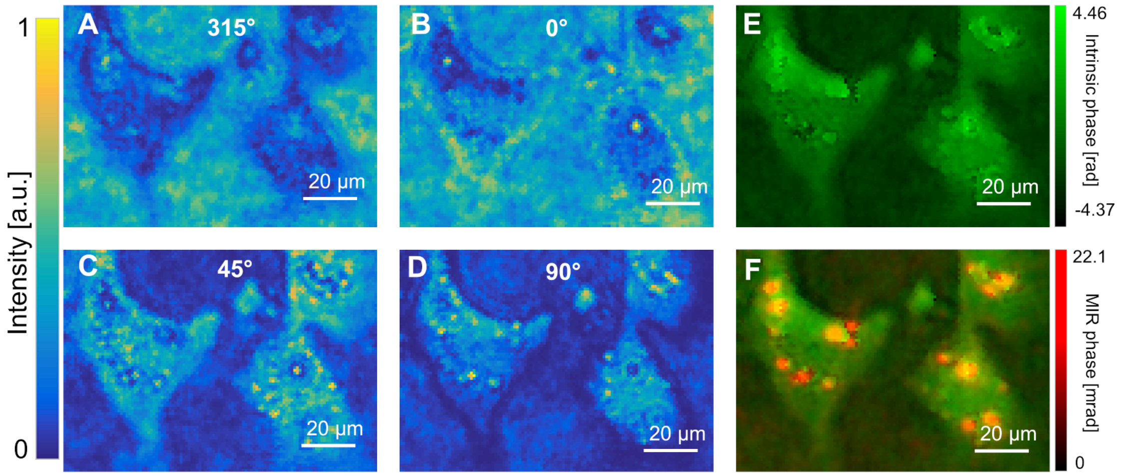

Fig. S9. Intensity images and quantitative intrinsic-phase of 3T3-L1 cells (same FOV as Fig. 2G,H). A-D, MIR-OFF intensity images of cells at analyzer angles of 315°, 0°, 45°, and 90°. **E**, Quantitative intrinsic-phase reconstructed from (A-D) using eq. S18. The quality guide phase unwrapping method was used for reconstructing the actual intrinsic-phase (*33, 34*). **F**, Merge of quantitative intrinsic-phase image and MIR-phase image at 2850 cm^{-1}.

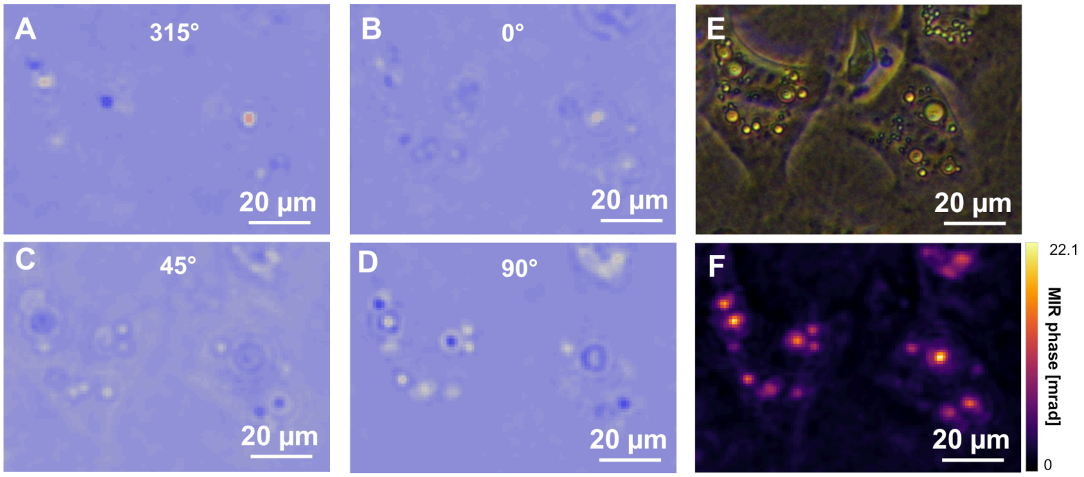

Fig. S10. Intensity subtraction images from measurements on live cells at a mid-IR wavenumber of 2850 cm^{-1}. **A-D**, Subtraction images for analyzer angles of 315°, 0°, 45°, and 90°. **E**, Image acquired by Zernike's phase-contrast microscope of the same FOV as (A-D). **F,** PSOM image constructed using eq. S11.

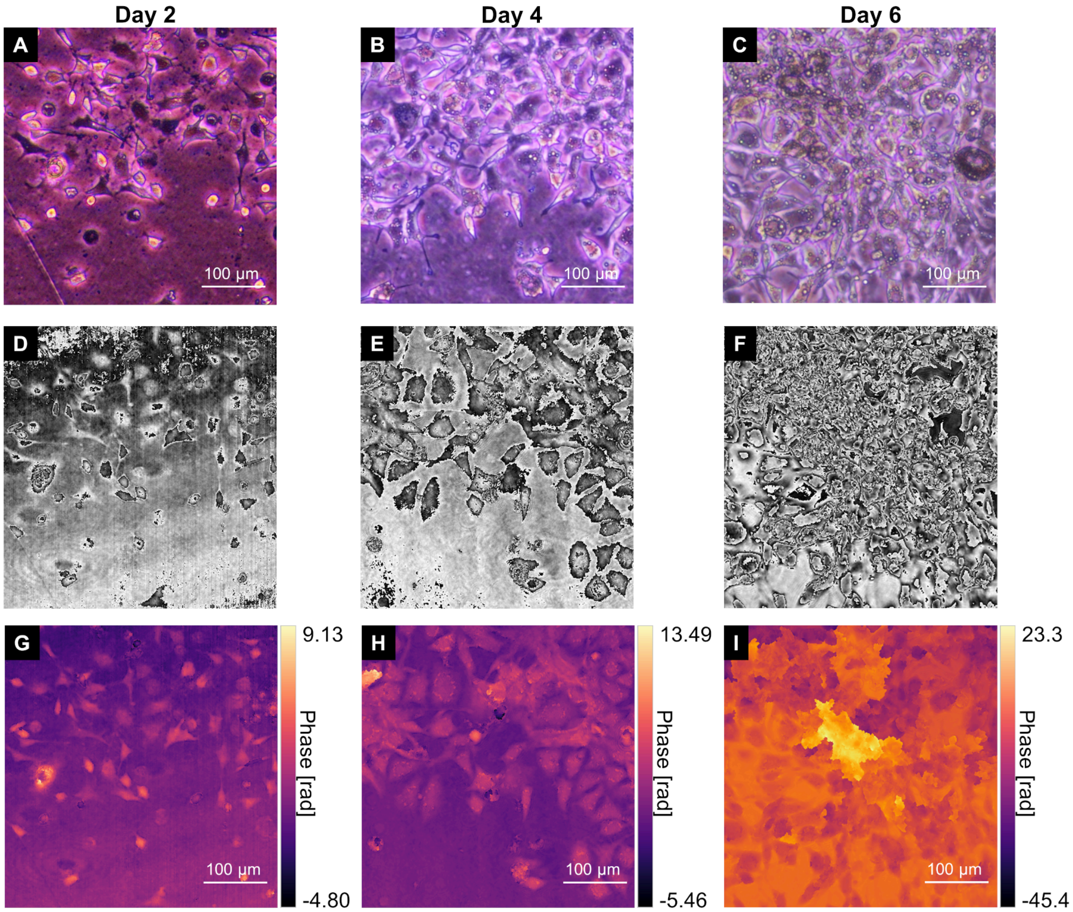

Fig. S11. Intrinsic-phase of 3T3-L1 cells on days 2, 4, and 6 after differentiation. A-C, Zernike phase contrast micrographs. **D-F,** Wrapped phase for the FOVs corresponding to (A-C) (applying eq. S18). **G-I,** Full range intrinsic-phase images reconstructed using a quality guide phase unwrapping method (*33, 34*). At high confluence (I), due to strong scattering caused by clumps of cells, the phase unwrapping method failed to recover morphological information.

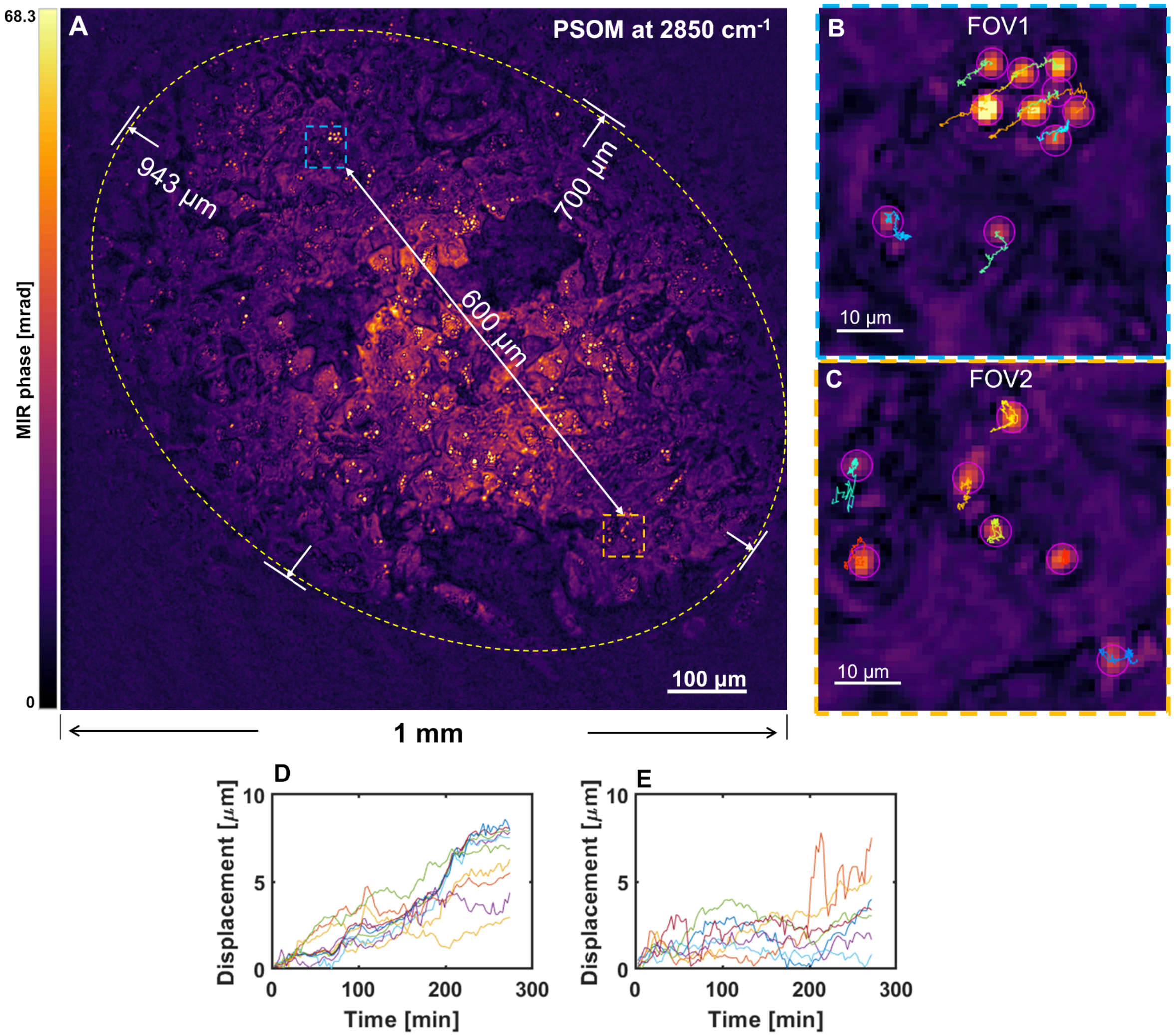

Fig. S12. Live-cell imaging on a large FOV by PSOM. A, Large FOV attained by expanding the mid-IR illumination spot used to measure dynamic lipid droplets (LDs) in living cells (3T3-L1). The effective FOV is highlighted by the dashed ellipse with major axis of 943 μm, and minor axis of 700 μm, corresponding to an FOV of 5.18×10^5 μm^2. The time taken to capture this image is 0.8 s. **B,C,** Two zoomed-in regions in FOV from (A), illustrating the paths of LDs tracked during a monitoring duration of 4.5 h. **D,E,** Plots of the LDs' displacement from their original locations, obtained from tracked LDs in (B) and (C) respectively.

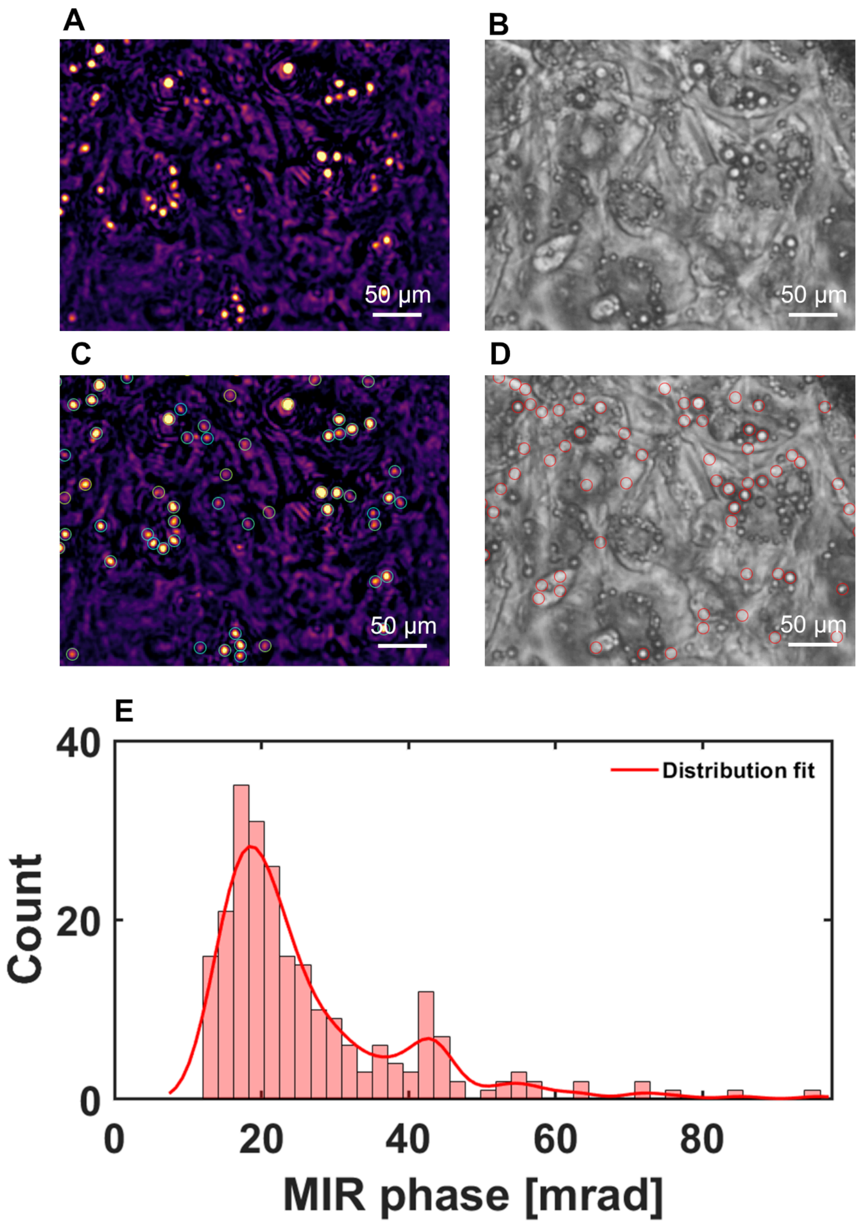

Fig. S13. Lipid droplet segmentations on PSOM and Zernike phase contrast (ZPC) images. A,B, Zoomed-in images of the dashed rectangles in the FOV of **Fig. 3A,B**. **C,D,** The detection program, TrackMate (*29, 30*), automatically marks lipid droplets (LDs) with circles. Using the same method, LDs in the PSOM micrograph (C) can be easily segmented from background, while LDs in the ZPC micrograph (D) are not segmented correctly due to strong phase background. **E,** Statistic analysis of the segmented lipid droplets obtained from the whole FOV of **Fig. 3B**, illustrating their MIR-phase distribution.

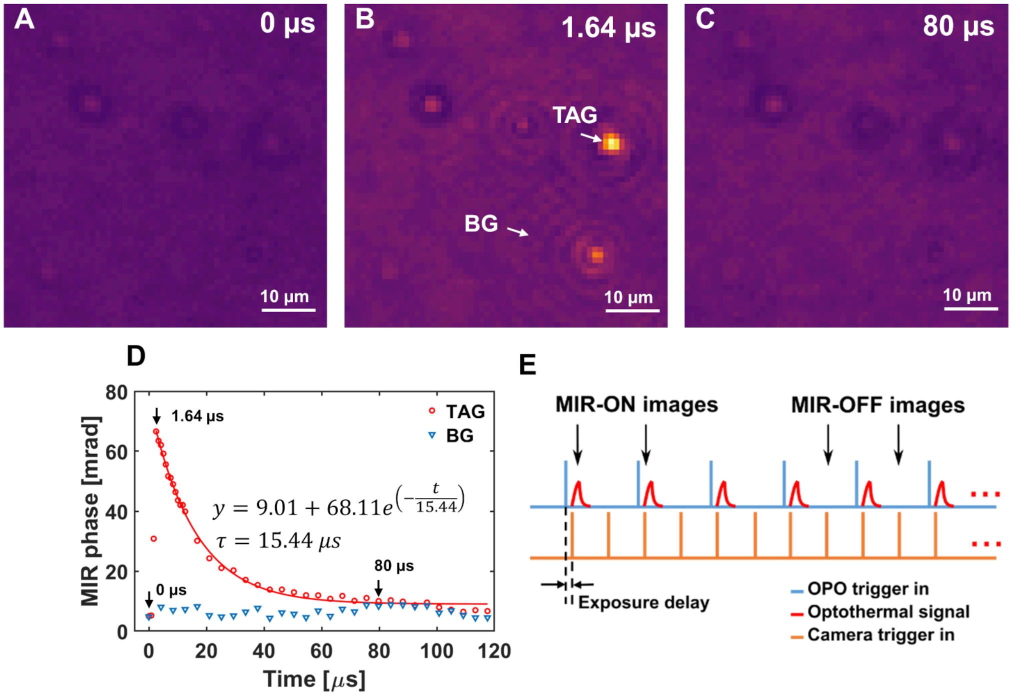

Fig. S14. Time-dependent optothermal transient signal measured from synthetic triglyceride (TAG) phantom. A-C, PSOM image of the FOV at exposure delay of 0 μs, 1.64 μs and 80 μs. **D,** Plot of MIR-phase values measured at a TAG drop and an area defined as background (BG) as marked in (B). **E,** The trigger pulse train used in this measurement. OPO: optical parametric oscillator.

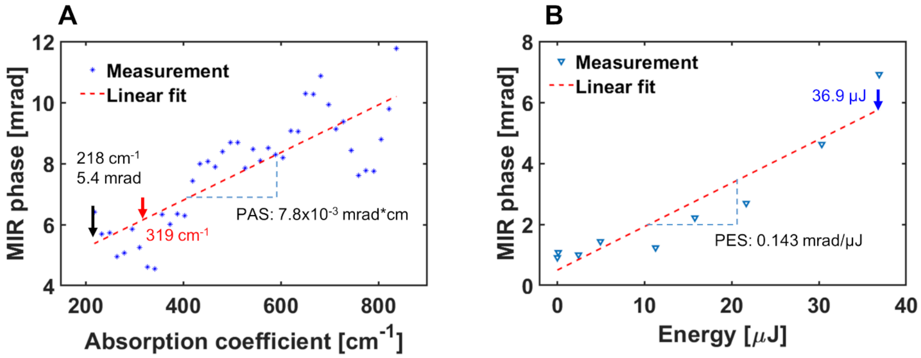

Fig. S15. MIR-phase sensitivity of PSOM for absorption coefficient and excitation energy in water. A, MIR-phase response vs. absorption coefficient at a pulse energy of 36.9 μJ. A phase-absorption sensitivity (PAS) constant of 7.8×10^{-3} mrad*cm was obtained from the linear fit of this measurement. **B,** MIR-phase response vs. mid-IR excitation energy for an absorption coefficient of 319 cm^{-1}. A phase-energy sensitivity (PES) constant of 0.143 mrad/μJ can be obtained from the linear fit of this measurement. The red arrow in (A) indicates the absorption coefficient of 319 cm^{-1} corresponding to the absorption coefficient for the measurement in (B). The blue arrow in (B) indicates the pulse energy (36.9 μJ) used for the measurement in (A). Values for the absorption coefficient of water were obtained from ref. (*35*).

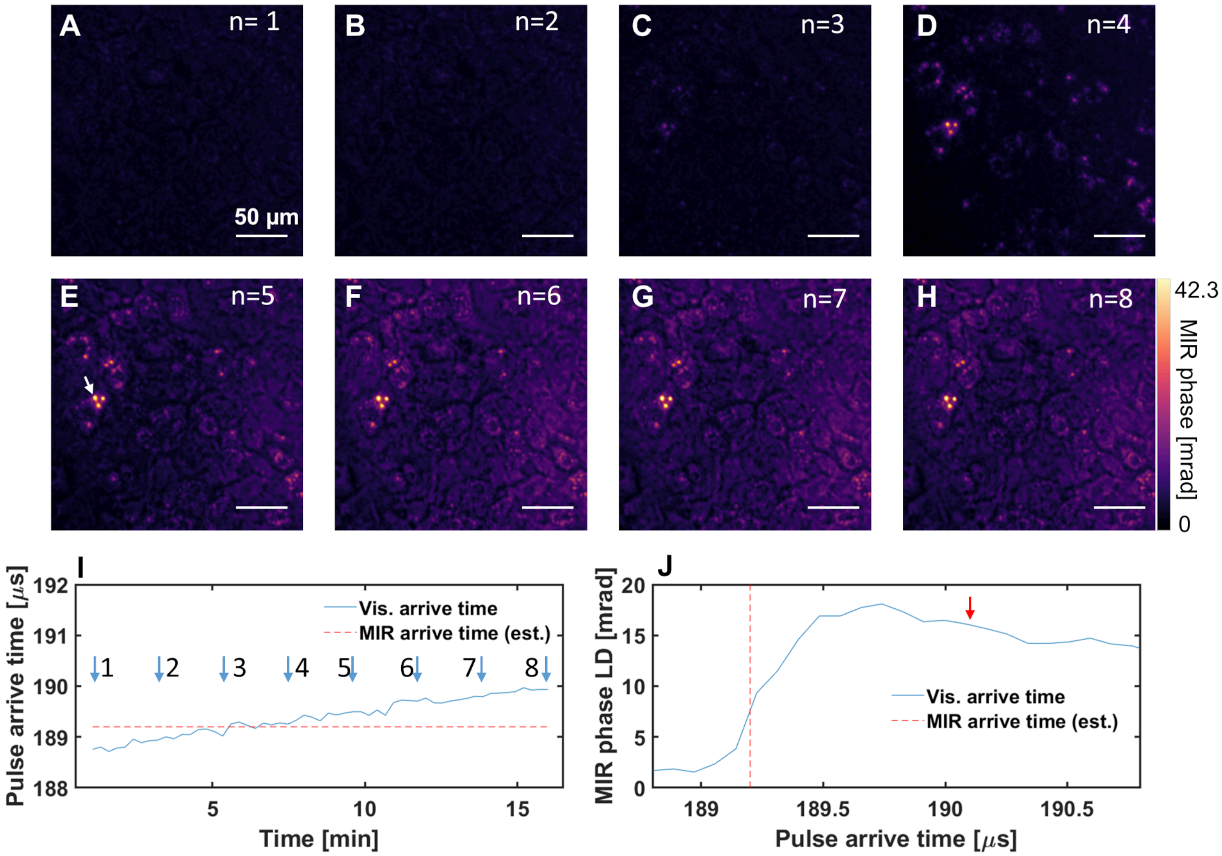

Fig. S16. Time-dependent optothermal transient signals measured from live cells. **A-H**, PSOM images acquired by varying the exposure delays (see **Materials & Methods**, and **Fig. S5**). **I**, The Vis. pulse arrival time measured by a photodiode detector and a synchronized data acquisition card (DAQ). The Vis. pulse arrival time for corresponding figures of (A-H) is marked by a number "1" to "8". **J,** Plot of MIR-phase vs. time for the LD marked in (E). Vis. arrive time, and MIR arrive time are defined as the time taken for the Vis. pulse to hit the sample plane, and the time taken for the mid-IR pulse to hit the sample plane after triggering the mid-IR. Vis.: visible laser; MIR: mid-infrared laser.

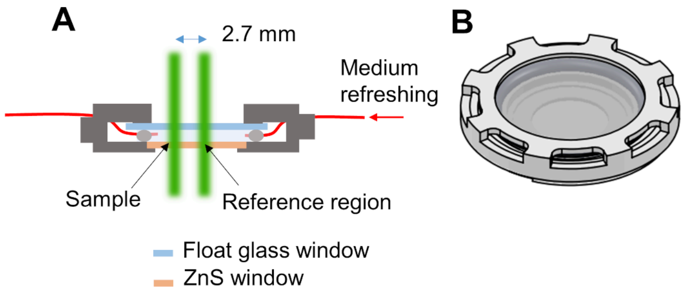

Fig. S17. Configuration for measuring living cells. **A**, Side view of the dish during the measurement of living cells. **B,** 3D view of the dish. The cells are maintained in a dish with two parallel windows (as seen in Panel A, bottom: ZnS window; top: Float glass window). The two probe beams 2.7 mm apart pass through the dish, with one beam passing through a sample region (with cells) and the other beam passing through a reference region (with no cells). For monitoring over a long duration, the medium can be refreshed through an inlet going into the dish.

Table S1. Comparison of PSOM with state-of-the-art optothermal/photothermal microscopy methods. Phase-absorption sensitivity (PAS) and phase-energy sensitivity (PES) for water were calculated in **Fig. S15**.

	**WOMiM** Based on values from Ref. (*16*)	**BSTP** Based on values from Ref. (*14*)	**ADRIFT** Based on values from Ref. (*20*)	**BS-IDT** Based on values from Ref. (*21*)	**PSOM**
FOV (μm^2)	2.5 ×10^4	~2.0×10^3	~2.0×10^3	1.0×10^4	5.2 ×10^5
Repetition rate (kHz)	1	150	1	10	1
MIR shot-noise (mrad)	Not provided (-)	~5	~ 1	Not Applicable (NA)	0.33
SNR	~50	30-100	-	-	203
Excitation irradiance/PFD ($\mu W/\mu m^2$)	0.39	30	0.06	~0.5	0.07
Resolution (μm)	1.85	0.96	-	0.35	2.03
Imaging speed (Frame per second)	50	1-100	5	0.05-6	0.017
High Confluence (Yes/No, y/n)?	n	n	n	n	y
Phase-wrapping artifacts (y/n)?	y	y	y	NA	n
Demonstrated cell viability (y/n)?	n	n	n	n	y
Living cells (y/n)?	n	y	y	n	y
Phase-absorption sensitivity, PAS (mrad*cm)	-	-	-	-	7.8×10^{-3}
Phase-energy sensitivity, PES (mrad/μJ)	-	-	-	-	0.143

Table S2. Comparison of focal excitation and wide-field excitation imaging methods. FOV: Field-of-view. (*11, 14, 28, 36*)

	**Focal excitation**		**Wide-field excitation**
	Galvo scanning	Mechanical-scanning	
Has trade-off between imaging time & FOV?	Yes	Yes	No
Excitation irradiance $\mu W/\mu m^2$)	1.5×10^4	8.0	0.05
Imaging speed (fps)	~10-30 (For FOV of c.a. 1×10^6 μm^2)	0.0055 (For FOV of c.a. 1×10^6 μm^2)	100 (Does not depend on FOV)

REFERENCES AND NOTES

1. M. D. Duncan, J. Reintjes, T. J. Manuccia, Scanning coherent anti-Stokes Raman microscope. *Opt. Lett.* **7**, 350–352 (1982).

2. A. Zumbusch, G. R. Holtom, X. S. Xie, Three-dimensional vibrational imaging by coherent anti-Stokes Raman scattering. *Phys. Rev. Lett.* **82**, 4142–4145 (1999).

3. C. W. Freudiger, W. Min, B. G. Saar, S. Lu, G. R. Holtom, C. He, J. C. Tsai, J. X. Kang, X. S. Xie, Label-free biomedical imaging with high sensitivity by stimulated Raman scattering microscopy. *Science* **322**, 1857–1861 (2008).

4. S. Kumar, A. Srinivasan, F. Nikolajeff, Role of infrared spectroscopy and imaging in cancer diagnosis. *Curr. Med. Chem.* **25**, 1055–1072 (2018).

5. S. Bégin, B. Burgoyne, V. Mercier, A. Villeneuve, R. Vallée, D. Côté, Coherent anti-Stokes Raman scattering hyperspectral tissue imaging with a wavelength-swept system. *Biomed. Opt. Express* **2**, 1296–1306 (2011).

6. D. Fu, G. Holtom, C. Freudiger, X. Zhang, X. S. Xie, Hyperspectral imaging with stimulated Raman scattering by chirped femtosecond lasers. *J. Phys. Chem. B* **117**, 4634–4640 (2013).

7. S. M. Burrows, R. D. Reif, D. Pappas, Investigation of photobleaching and saturation of single molecules by fluorophore recrossing events. *Anal. Chim. Acta* **598**, 135–142 (2007).

8. D. A. Helmerich, G. Beliu, S. S. Matikonda, M. J. Schnermann, M. Sauer, Photoblueing of organic dyes can cause artifacts in super-resolution microscopy. *Nat. Methods* **18**, 253–257 (2021).

9. N. Fili, C. P. Toseland, Fluorescence and labelling: How to choose and what to do. *Exo. Supppl.* **105**, 1–24 (2014).

10. A. Schwaighofer, M. Brandstetter, B. Lendl, Quantum cascade lasers (QCLs) in biomedical spectroscopy. *Chem. Soc. Rev.* **46**, 5903–5924 (2017).

11. C. L. Evans, E. O. Potma, M. Puoris'haag, D. Côté, C. P. Lin, X. S. Xie, Chemical imaging of tissue in vivo with video-rate coherent anti-Stokes Raman scattering microscopy. *Proc. Natl. Acad. Sci. U.S.A.* **102**, 16807–16812 (2005).

12. L. Kong, M. Navas-Moreno, J. W. Chan, Fast confocal Raman imaging using a 2-D multifocal array for parallel hyperspectral detection. *Anal. Chem.* **88**, 1281–1285 (2016).

13. M. J. Nasse, M. J. Walsh, E. C. Mattson, R. Reininger, A. Kajdacsy-Balla, V. Macias, R. Bhargava, C. J. Hirschmugl, High-resolution Fourier-transform infrared chemical imaging with multiple synchrotron beams. *Nat. Methods* **8**, 413–416 (2011).

14. D. Zhang, L. Lan, Y. Bai, H. Majeed, M. E. Kandel, G. Popescu, J.-X. Cheng, Bond-selective transient phase imaging via sensing of the infrared photothermal effect. *Light Sci. Appl.* **8**, 116 (2019).

15. M. Tamamitsu, K. Toda, H. Shimada, T. Honda, M. Takarada, K. Okabe, Y. Nagashima, R. Horisaki, T. Ideguchi, Label-free biochemical quantitative phase imaging with mid-infrared photothermal effect. *Optica* **7**, 359–366 (2020).

16. T. Yuan, M. A. Pleitez, F. Gasparin, V. Ntziachristos, Wide-field mid-infrared hyperspectral imaging by snapshot phase contrast measurement of optothermal excitation. *Anal. Chem.* **93**, 15323–15330 (2021).

17. T. Ikeda, G. Popescu, R. R. Dasari, M. S. Feld, Hilbert phase microscopy for investigating fast dynamics in transparent systems. *Opt. Lett.* **30**, 1165–1167 (2005).

18. B. Bhaduri, G. Popescu, Derivative method for phase retrieval in off-axis quantitative phase imaging. *Opt. Lett.* **37**, 1868–1870 (2012).

19. X. Xie, Iterated unscented Kalman filter for phase unwrapping of interferometric fringes. *Opt. Express* **24**, 18872–18897 (2016).

20. K. Toda, M. Tamamitsu, T. Ideguchi, Adaptive dynamic range shift (ADRIFT) quantitative phase imaging. *Light Sci. Appl.* **10**, 1 (2021).

21. J. Zhao, A. Matlock, H. Zhu, Z. Song, J. Zhu, B. Wang, F. Chen, Y. Zhan, Z. Chen, Y. Xu, X. Lin, L. Tian, J.-X. Cheng, Bond-selective intensity diffraction tomography. *Nat. Commun.* **13**, 7767 (2022).

22. S. Murugkar, C. Brideau, A. Ridsdale, M. Naji, P. K. Stys, H. Anis, Coherent anti-Stokes Raman scattering microscopy using photonic crystal fiber with two closely lying zero dispersion wavelengths. *Opt. Express* **15**, 14028–14037 (2007).

23. L. M. Riobó, F. E. Veiras, P. A. Sorichetti, M. T. Garea, Wideband quad optical sensor for high-speed sub-nanometer interferometry. *Appl. Opt.* **56**, 397–403 (2017).

24. L. Riobó, Y. Hazan, F. Veiras, M. Garea, P. Sorichetti, A. Rosenthal, Noise reduction in resonator-based ultrasound sensors by using a CW laser and phase detection. *Opt. Lett.* **44**, 2677–2680 (2019).

25. G. Topman, O. Sharabani-Yosef, A. Gefen, A method for quick, low-cost automated confluency measurements. *Microsc. Microanal.* **17**, 915–922 (2011).

26. H. Salehi, L. Derely, A.-G. Vegh, J.-C. Durand, C. Gergely, C. Larroque, M.-A. Fauroux, F. J. Cuisinier, Label-free detection of anticancer drug paclitaxel in living cells by confocal Raman microscopy. *Appl. Phys. Lett.* **102**, 113701 (2013).

27. X. Wu, M. Pankow, T. Onuma, H.-Y. S. Huang, K. Peters, Comparison of high-speed polarization imaging methods for biological tissues. *Sensors* **22**, 8000 (2022).

28. P. Fu, W. Cao, T. Chen, X. Huang, T. Le, S. Zhu, D.-W. Wang, H. J. Lee, D. Zhang, Super-resolution imaging of non-fluorescent molecules by photothermal relaxation localization microscopy. *Nat. Photonics* **17**, 330–337 (2023).

29. K. Jaqaman, D. Loerke, M. Mettlen, H. Kuwata, S. Grinstein, S. L. Schmid, G. Danuser, Robust single-particle tracking in live-cell time-lapse sequences. *Nat. Methods* **5**, 695–702 (2008).

30. D. Ershov, M.-S. Phan, J. W. Pylvänäinen, S. U. Rigaud, L. L. Blanc, A. Charles-Orszag, J. R. W. Conway, R. F. Laine, N. H. Roy, D. Bonazzi, G. Duménil, G. Jacquemet, J.-Y. Tinevez,

TrackMate 7: Integrating state-of-the-art segmentation algorithms into tracking pipelines. *Nat. Methods* **19**, 829–832 (2022).

31. Y. Park, C. Depeursinge, G. Popescu, Quantitative phase imaging in biomedicine. *Nat. Photonics* **12**, 578–589 (2018).

32. C. Wang, Q. Fu, X. Dun, W. Heidrich, Quantitative phase and intensity microscopy using snapshot white light wavefront sensing. *Sci. Rep.* **9**, 13795 (2019).

33. M. Zhao, L. Huang, Q. Zhang, X. Su, A. Asundi, Q. Kemao, Quality-guided phase unwrapping technique: Comparison of quality maps and guiding strategies. *Appl. Opt.* **50**, 6214–6224 (2011).

34. M. Zhao, H. Wang, Q. Kemao, Snake-assisted quality-guided phase unwrapping for discontinuous phase fields. *Appl. Opt.* **54**, 7462–7470 (2015).

35. J. E. Bertie, Z. Lan, Infrared intensities of liquids XX: The intensity of the OH stretching band of liquid water revisited, and the best current values of the optical constants of H_2O(l) at 25°C between 15,000 and 1 cm^{-1}. *Appl. Spectrosc.* **50**, 1047–1057 (1996).

36. M. A. Pleitez, A. A. Khan, A. Soldà, A. Chmyrov, J. Reber, F. Gasparin, M. R. Seeger, B. Schätz, S. Herzig, M. Scheideler, V. Ntziachristos, Label-free metabolic imaging by mid-infrared optoacoustic microscopy in living cells. *Nat. Biotechnol.* **38**, 293–296 (2020).
